# Prolonged microgravity induces reversible and persistent changes on human cerebral connectivity

**DOI:** 10.1038/s42003-022-04382-w

**Published:** 2023-01-13

**Authors:** Steven Jillings, Ekaterina Pechenkova, Elena Tomilovskaya, Ilya Rukavishnikov, Ben Jeurissen, Angelique Van Ombergen, Inna Nosikova, Alena Rumshiskaya, Liudmila Litvinova, Jitka Annen, Chloë De Laet, Catho Schoenmaekers, Jan Sijbers, Victor Petrovichev, Stefan Sunaert, Paul M. Parizel, Valentin Sinitsyn, Peter zu Eulenburg, Steven Laureys, Athena Demertzi, Floris L. Wuyts

**Affiliations:** 1 Lab for Equilibrium Investigations and Aerospace, University of Antwerp, Antwerp, Belgium; 2 Laboratory for Cognitive Research, HSE University, Moscow, Russia; 3 SSC RF—Institute for Biomedical Problems, Russian Academy of Sciences, Moscow, Russia; 4 imec-Vision Lab, University of Antwerp, Antwerp, Belgium; 5 Department of Translational Neuroscience—ENT, University of Antwerp, Antwerp, Belgium; 6 Radiology Department, National Medical Research Treatment and Rehabilitation Center of the Ministry of Health of Russia, Moscow, Russia; 7 Coma Science Group, GIGA Consciousness, GIGA Institute, University and University Hospital of Liège, Liège, Belgium; 8 Department of Imaging & Pathology, Translational MRI, KU Leuven—University of Leuven, Leuven, Belgium; 9 Department of Radiology, Royal Perth Hospital and University of Western Australia Medical School, Perth, WA Australia; 10 Faculty of Fundamental Medicine, Lomonosov Moscow State University, Moscow, Russia; 11 Institute for Neuroradiology, Ludwig-Maximilians-University Munich, Munich, Germany; 12 Joint International Research Unit on Consciousness, CERVO Brain Research Centre, Laval University, Quebec, QC Canada; 13 International Consciousness Science Institute, Hangzhou Normal University, Hangzhou, China; 14 Physiology of Cognition, GIGA-CRC In Vivo Imaging, University of Liège, Liège, Belgium; 15 Department of Psychology, Psychology and Neuroscience of Cognition Research Unit, University of Liège, Liège, Belgium

**Keywords:** Neuroscience, Stress and resilience

## Abstract

The prospect of continued manned space missions warrants an in-depth understanding of how prolonged microgravity affects the human brain. Functional magnetic resonance imaging (fMRI) can pinpoint changes reflecting adaptive neuroplasticity across time. We acquired resting-state fMRI data of cosmonauts before, shortly after, and eight months after spaceflight as a follow-up to assess global connectivity changes over time. Our results show persisting connectivity decreases in posterior cingulate cortex and thalamus and persisting increases in the right angular gyrus. Connectivity in the bilateral insular cortex decreased after spaceflight, which reversed at follow-up. No significant connectivity changes across eight months were found in a matched control group. Overall, we show that altered gravitational environments influence functional connectivity longitudinally in multimodal brain hubs, reflecting adaptations to unfamiliar and conflicting sensory input in microgravity. These results provide insights into brain functional modifications occurring during spaceflight, and their further development when back on Earth.

## Abstract (web summary)

rs-fMRI is used to investigate human brain connectivity changes induced by prolonged microgravity in cosmonauts before and after spaceflight, with both persistent and reversible location specific changes in connectivity being observed.

## Introduction

The human brain is highly adaptive to exogenous and endogenous changes, a process known as neuroplasticity^1^. Neuroplasticity has primarily concerned adaptations of the brain’s function and structure during development^2^, after brain damage^3^, and during skill learning^4^. Yet, little is known about how our brains can cope with extreme environmental factors, such as changes in gravitational forces. This question is particularly pertinent when one considers long-term spaceflight missions to the International Space Station (ISS), or future missions to the Moon and Mars, during which humans need to endure exposure to extreme conditions for prolonged periods of time.

In terms of brain anatomy, comparing structural magnetic resonance imaging (MRI) data from space travelers before and after long-duration missions showed a redistribution of the subarachnoid cerebrospinal fluid (CSF)^4–7^, ventricular enlargement^8–10^, and macroscopic gray matter changes indicative of shape changes and remodeling^5,6,11^. A large part of these modifications is ascribed to the upward shift of bodily fluids occurring upon entering a microgravity environment^5–8,10,11^. Also, a net increase of neural tissue in several sensorimotor brain areas have been observed, such as in the cerebellum and basal ganglia. These findings strongly point toward neuroplastic mechanisms indicative of motor strategy adaptation^6^. Importantly, most of such fluid shift-driven structural brain changes appear to partially persist for at least 7 months, as shown in a follow-up postflight scanning session^5,6,10^, with ventricular volume persisting up to a year after the mission^12^. These findings emphasize the need to further research the dynamics of spaceflight-induced brain changes for a prolonged period after the space mission.

At the functional level, a single cosmonaut study using resting-state functional MRI (fMRI) found that vestibular (insula) and motor regions showed reduced participation in whole-brain functional connectivity^13^. Altering gravity levels induced with parabolic flight was found to decrease connectivity in the right temporo-parietal junction in healthy volunteers using resting-state fMRI scans acquired before and after the flight session^14^. These results suggest that multisensory regions might mediate the neuroplastic adaptations to gravity alterations^14^. In one group-level fMRI study in cosmonauts, the somatosensory input that is experienced during walking was mimicked by stimulating the foot soles of the cosmonauts during scanning. This study reported various functional connectivity changes after long-duration spaceflight in multisensory integration areas, including right supramarginal gyrus, posterior and anterior insular cortex, and angular gyrus^15^. More recently, there has been evidence for sensory reweighting at the cortical level in response to vestibular stimulation induced by skull tapping, i.e., postflight increases in visual and somatosensory activations^16^. The latter study also showed that this adaptive response to spaceflight recovered after 3 months postflight.

Altogether, these studies provide evidence on functional changes in vestibular, motor, and multisensory brain regions induced by gravity alterations, which may well reflect the brain’s adaptive processes to cope with altered sensorimotor demands in these environmental conditions. Given that structural MRI mainly reveals brain changes after spaceflight that relate to fluid and brain shifts, fMRI can play an important role of better pinpointing changes reflecting adaptive neuroplasticity. We here aim to provide evidence for longitudinal functional connectivity adaptations in a cohort of cosmonauts by repeatedly collecting resting-state fMRI data before and twice after their mission to the ISS. We specifically sought to characterize functional connectivity changes after spaceflight which were either sustained or were normalized to baseline at the follow-up session more than a half year after their mission. We adopted a whole-brain exploratory approach with the main aim to map out which different brain regions are functionally modulated by long-duration spaceflight, irrespective of their association with behavior and performance, considering the lack of access to such data.

## Results

Overall, we found that exposure to prolonged microgravity influenced longitudinal functional connectivity in a heterogeneous manner, decreasing the overall connectivity in some areas and increasing it in others.

### Connectivity modulations after long-duration spaceflight

**Fig. 1:**
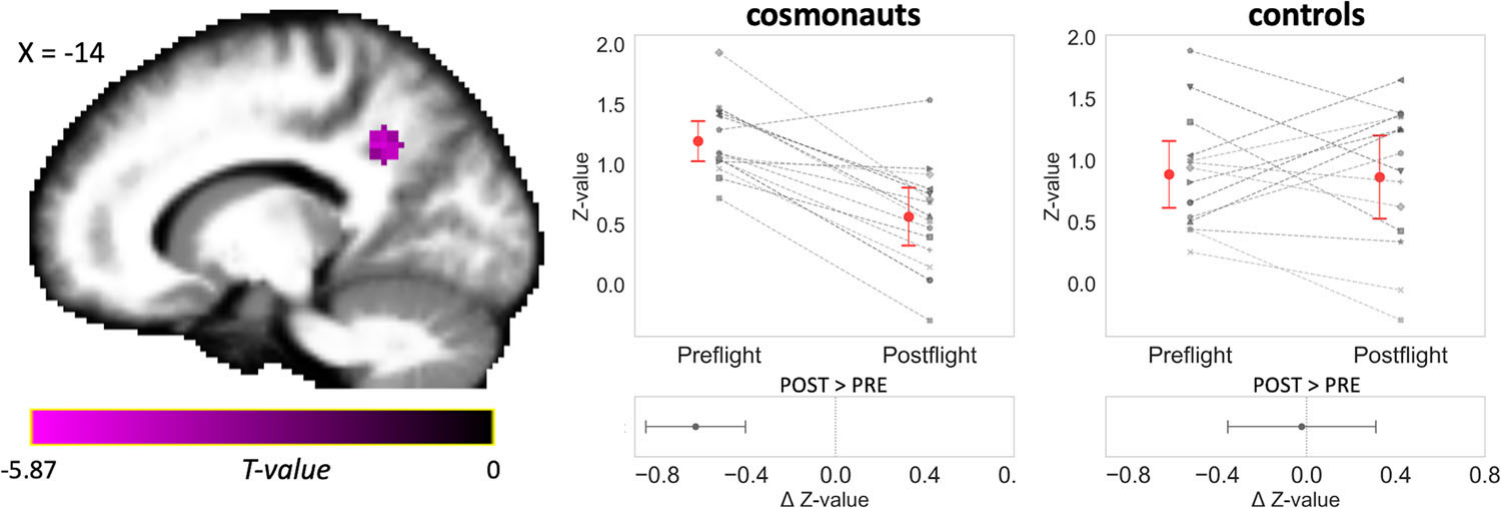
Connectivity of the posterior cingulate cortex with the rest of the brain is reduced after spaceflight. At postflight, cosmonauts exhibited decreased participation of the posterior cingulate cortex (PCC) in whole-brain connectivity when compared to the preflight scan. Individual intrinsic connectivity contrast (ICC) values (gray) as extracted from the PCC cluster across the two scans in the cosmonaut group confirmed this decreasing effect for most cosmonauts (red: mean). For comparative purposes, in the control group (*n* = 14) ICC values did not show significant modifications across time (average change approximated zero). Subplots summarize the estimated differences between the two timepoints. Error bars indicate 95% confidence intervals. Slice coordinates are in MNI space. Statistical significance is based on *p* < 0.005 uncorrected at the voxel level followed by *p* < 0.05 corrected for family-wise error at the cluster level (*n* = 15).

As a sanity check, we first compared two connectivity datasets acquired in a control group more than half a year apart and using the same MRI device and protocol as the cosmonauts. We found no significant changes in intrinsic connectivity contrast (ICC) over time, with ICC being a voxel-based measure for global connectivity. We then compared postflight ICC values in cosmonauts to their preflight values and found an ICC decrease in a cluster covering the left precuneus and posterior cingulate cortex (PCC) postflight (Fig. 1, Table 1). These results were further confirmed by Bayesian statistics, indicating that the obtained effects in the cosmonaut cohort can be attributed to prolonged microgravity more confidently. Specifically, there was extreme evidence for a change in time in cosmonauts (BF_10_ (error) = 826 (6.98^−7^)), while there was no such evidence in the control group (BF_10_ (error) = 0.272 (0.008)). The interaction test also revealed that the pre- to postflight change in cosmonauts differed from the effect over time in controls (BF_10_ (error) = 14.124 (8.81^−6^)).

**Table 1:** Cluster information of the global connectivity analysis results in cosmonauts.

Contrast/Brain region		Peak voxel coordinates			cluster size	beta	T-value	*p*-cFWE
		x	y	z				
**Postflight** **>** **Preflight [−1 1] (df** **=** **14)**								
No significant results								
**Preflight** **>** **Postflight [1 −1] (df** **=** **14)**								
Precuneus/Posterior cingulate cortex, L		−12	−52	34	146	0.63	6.04	0.012
**Sustain model, increases [−1 0.5 0.5] (df** **=** **10)**								
Angular gyrus/Supramarginal gyrus, R		54	−46	46	104	0.59	7.13	0.042
**Sustain model, decrease [1 −0.5 −0.5] (df** **=** **10)**								
Precuneus/posterior cingulate cortex, L		−12	−52	34	123	0.61	6.62	0.016
Thalamus, L		00	−20	10	117	0.48	8.92	0.021
**Normalize model, increases [−0.5 1 −0.5] (df** **=** **10)**								
No significant results								
**Normalize model, decreases [0.5 −1 0.5] (df** = **10)**								
Posterior Insular cortex, L		−36	−16	00	169	0.60	7.26	0.003
Anterior Insular cortex, R		48	02	−06	140	0.62	6.73	0.011

Peak voxels of clusters showing modulations in ICC values between various contrasts among the three scanning sessions in the cosmonaut cohort (preflight, postflight, follow-up). Brain region labeling was based on the Neuromorphometrics atlas. Beta values represent the effect size; T-values are cluster-averaged; *p*-cFWE: *p*-value after cluster-wise family-wise error (FWE) correction. This threshold was set at *p* < 0.05. df = degrees of freedom. R = right, L = Left. Bold fonts are used as headers to define the exact test that was performed.

### Connectivity modulations sustain or normalize when back on Earth

**Fig. 2:**
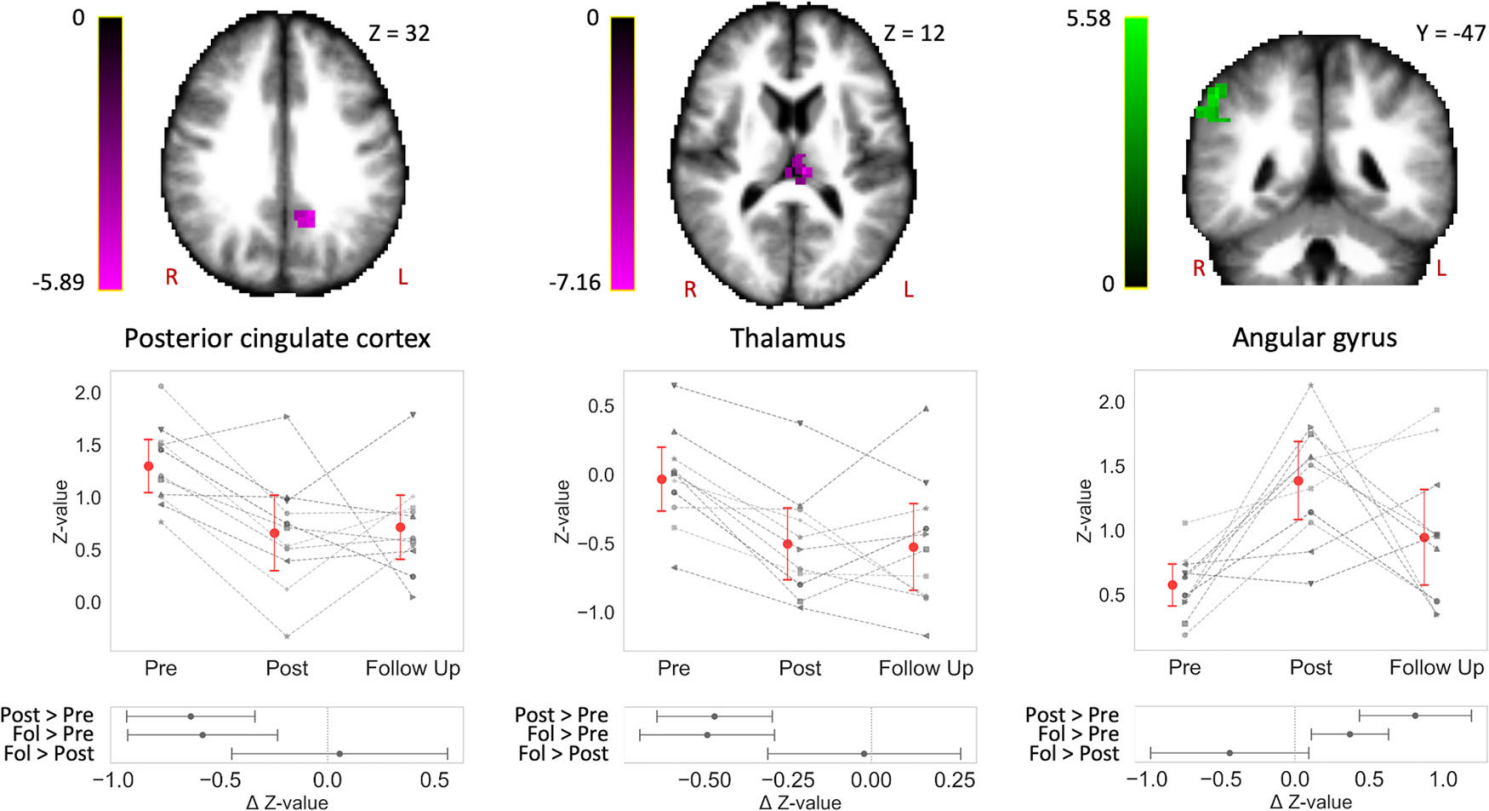
Connectivity in posterior cingulate cortex, thalamus, and right angular gyrus is changed after spaceflight and sustains in the longer term. The posterior cingulate cortex and the thalamus showed decreased participation in whole-brain connectivity (magenta), while the right angular gyrus exhibited increased participation in whole-brain connectivity (green) at postflight compared to preflight. These effects were found to persist up to 8 months after the space mission (Fol for follow-up). Significant clusters are scaled by *t*-statistic and slice coordinates are represented in MNI space. The plots illustrate cosmonaut-specific (gray) intrinsic connectivity contrast (ICC) changes in each significant cluster (red: mean). Subplots summarize the estimated differences between pairs of timepoints. Error bars indicate 95% confidence intervals. Statistical significance is based on *p* < 0.005 uncorrected at the voxel level followed by *p* < 0.05 corrected for family-wise error at the cluster level (*n* = 11). R right, L left.

**Fig. 3:**
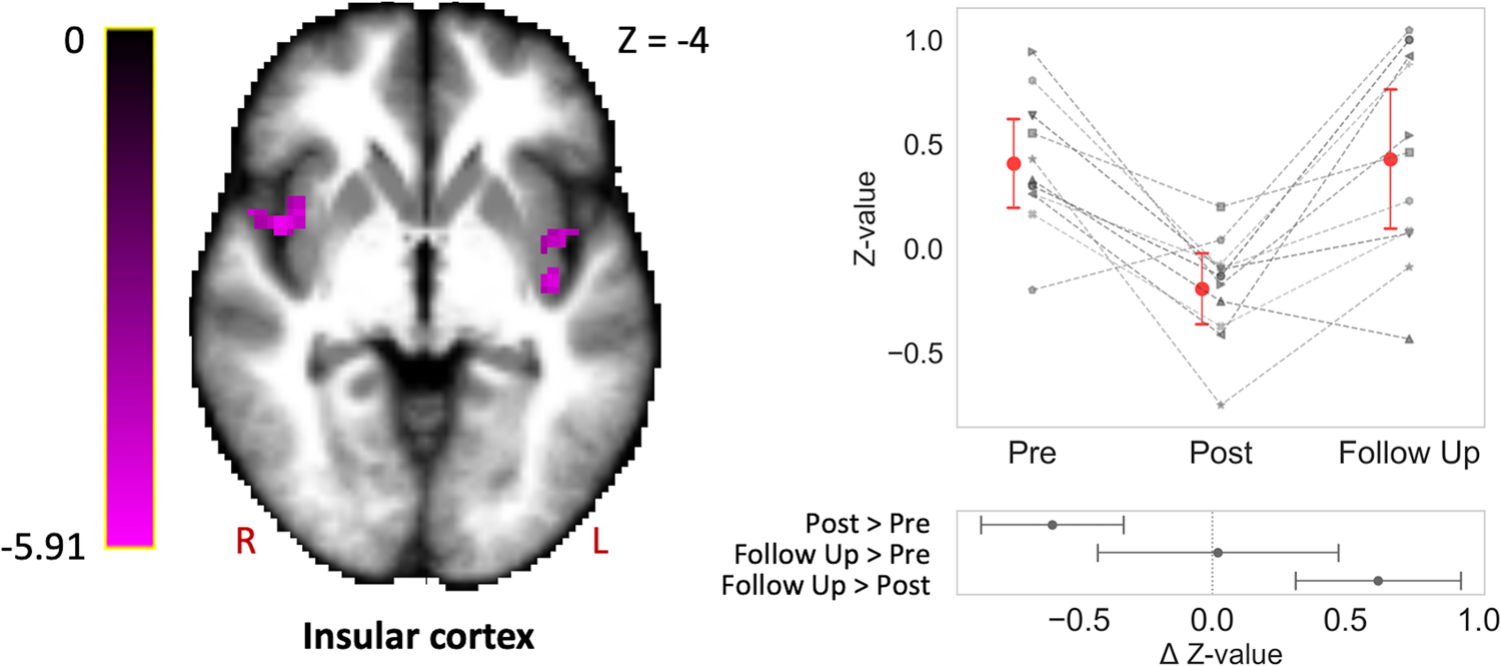
Connectivity in bilateral insular cortex is decreased after spaceflight and normalizes in the long-term. The bilateral insular cortex showed decreased participation in whole-brain connectivity at postflight compared to preflight, which normalized back to preflight levels 8 months after return from space. Significant clusters are scaled by *t*-statistic and slice coordinates are represented in MNI space. The plot illustrates cosmonaut-specific (gray) intrinsic connectivity contrast (ICC) changes in the insular cluster (red: mean). Subplots summarize the estimated difference between pairs of timepoints. Error bars indicate 95% confidence intervals. Statistical significance is based on *p* < 0.005 uncorrected at the voxel level followed by *p* < 0.05 corrected for family-wise error at the cluster level (*n* = 11). R right, L left.

Next, we tested for connectivity changes from pre- to postflight, which then either sustained from postflight to follow-up (i.e., 8 months postflight) or either normalized back to preflight levels at follow-up. For the sustained longitudinal effects, we found postflight ICC decreases in the left precuneus/PCC and the left thalamus, which remained at follow-up. Additionally, ICC increases were found in a cluster overlapping the right angular gyrus and the supramarginal gyrus, which further remained at follow-up (Fig. 2, Table 1). For the normalized longitudinal effects, we found postflight ICC decreases in bilateral insular cortex, which had follow-up values comparable to the preflight data (Fig. 3, Table 1). Both clusters were located at the transition between anterior and posterior regions of the insular cortex. Again, Bayesian analyses were performed using the preflight and postflight data in cosmonauts, as well as the matched data in the control group. This revealed very strong to extreme evidence for pre- to postflight ICC changes in the PCC, thalamus, angular gyrus, and insula in the cosmonaut group, whereas there was no such evidence in the control group. The interaction test also showed moderate to strong evidence that the pre- to postflight changes in cosmonauts were different from the changes over time in the control group. Detailed results of the Bayesian analyses are found in the Supplementary Table 1.

### Network associations of brain regions showing connectivity changes after spaceflight

**Fig. 4:**
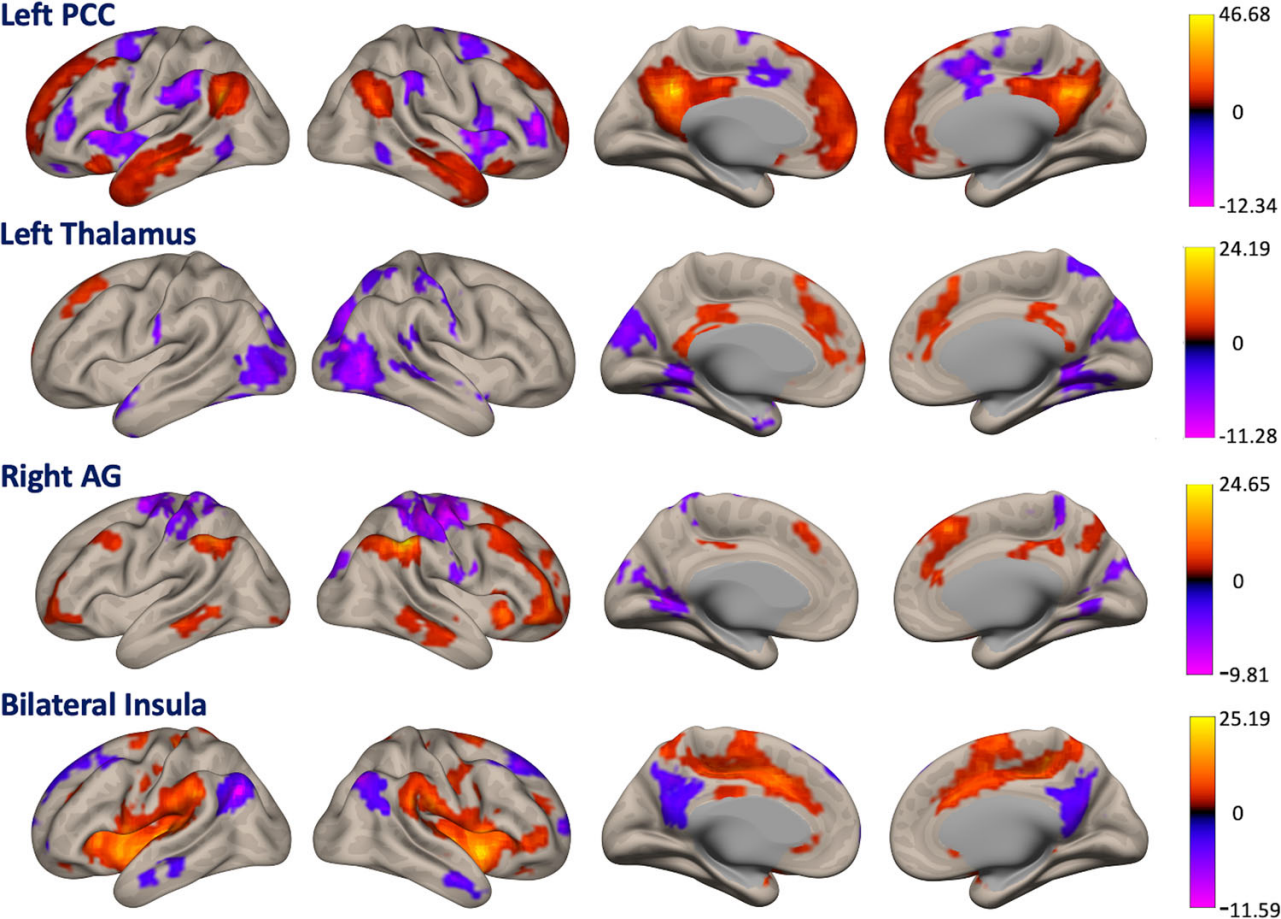
Functional connectivity networks associated with the posterior cingulate cortex, thalamus, right angular gyrus, and bilateral insula. In a seed correlation analysis, those regions that showed intrinsic connectivity contrast (ICC) modifications were used as seed areas to evaluate the ensuing brain networks. The posterior cingulate cortex and insula are associated with established default mode network and salience network regions, respectively. Color bars represent the *t*-values for the one-sample *t*-test. A statistical uncorrected voxel-level threshold of *p* < 0.001 at the whole-brain level was applied, with a subsequent cluster-level threshold of *p* < 0.05 family-wise error corrected (*n* = 11).

For each of the emerging clusters from the ICC analysis, we mapped the positive and negative correlations between the respective cluster and the rest of the brain to assess correspondence with known resting-state networks (Fig. 4). We found that the PCC belonged to the default mode network (DMN), as it is positively correlated with the medial prefrontal cortex, bilateral inferior parietal lobules, and bilateral anterior temporal lobes, while it is anti-correlated with lateral frontal and parietal regions and the anterior insular cortex, typically belonging to the executive and attention networks. The thalamus showed positive correlations with medial and superior frontal regions and cingulate cortex, although no network label could be assigned to this set of brain regions. Anti-correlations were found with the visual network, including occipital, temporal, and parietal regions. The right angular gyrus exhibited positive correlations with lateral prefrontal cortex, bilateral middle temporal gyrus, left angular gyrus, and right anterior insula. Altogether, there was a predominant overlap with the control network. Anti-correlations were found between the right angular gyrus and the sensorimotor cortex in the pre- and postcentral gyri, and occipital lobe. Lastly, the insular cortex exhibited positive correlation with the anterior to middle cingulate cortex and the supramarginal gyrus bilaterally, largely known as the salience or ventral attention network. Anti-correlations were found between the bilateral insula and the PCC, the angular gyrus bilaterally, superior frontal cortex and anterior temporal lobe.

### Global connectivity changes are associated with specific region-to-region connectivity changes

**Fig. 5:**
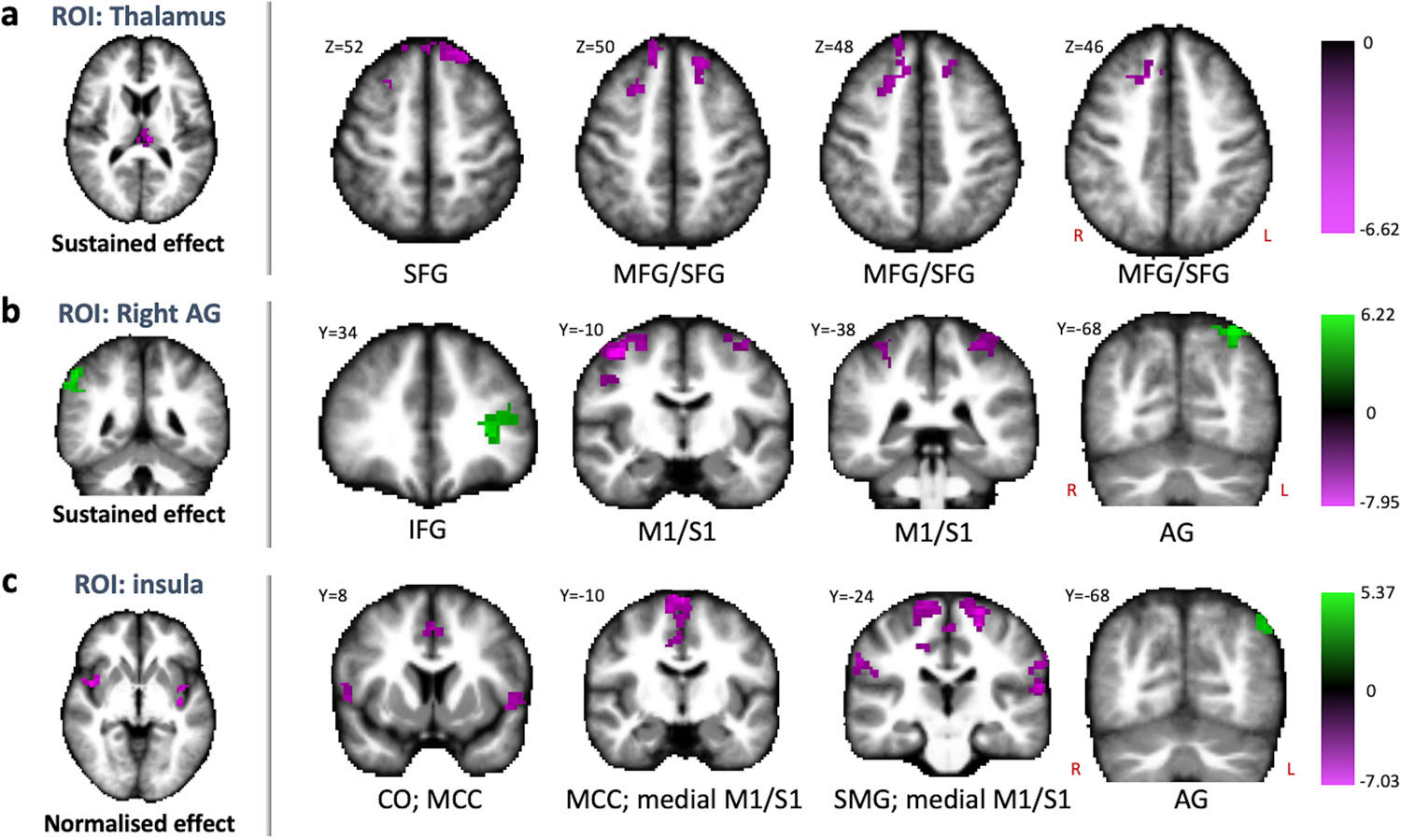
Seed-based connectivity changes after spaceflight of the thalamus, right angular gyrus, and bilateral insula. The longitudinal changes in intrinsic connectivity contrast (ICC) for the thalamus (**a**), right angular gyrus (**b**), and bilateral insula (**c**) were associated with connectivity increases (green) and decreases (magenta) with specific brain regions. The posterior cingulate cortex did not exhibit connectivity changes with other specific brain regions. Slice coordinates are presented in MNI space. Color maps present statistical *t*-values. AG angular gyrus, CO central operculum, IFG inferior frontal gyrus, M1/S1 pre- and postcentral gyrus, MCC middle cingulate cortex, MFG middle frontal gyrus, ROI region of interest, SFG superior frontal gyrus. SMG supramarginal gyrus. R right, L left. Statistical significance is based on *p* < 0.005 uncorrected at the voxel level followed by *p* < 0.05 corrected for family-wise error at the cluster level (*n* = 11).

Finally, we performed a seed-to-voxel connectivity analysis, using the emerging clusters from the ICC analysis as seed regions. The aim of this analysis was to investigate which specific region-to-region connectivity changes over time contributed to the observed global connectivity changes. The seed-to-voxel analysis showed no significant connectivity changes between the PCC and the rest of the brain, both when comparing postflight to preflight and when performing the statistical test for sustained effects. Results for the thalamus, though, showed that the decreased ICC that sustained over time was mostly due to a sustained decrease in postflight connectivity with the right middle frontal gyrus and the left superior frontal gyrus (Fig. 5, Table 2). Similarly, results for the right angular gyrus showed that the sustained high ICC values in that region was mostly due to a sustained decrease in connectivity between this region and bilateral pre- and postcentral gyri, as well as a sustained increase in connectivity with the left inferior frontal gyrus and left angular gyrus (Fig. 5, Table 2). Results for the bilateral insula showed that connectivity with middle cingulate cortex, bilateral pre- and postcentral gyri, central operculum bilaterally, and bilateral temporo-parietal junction (encompassing supramarginal gyrus and superior temporal gyrus) decreased postflight and normalized (i.e., increased) from postflight to follow-up (Fig. 5, Table 2). Additionally, the bilateral insula showed a postflight connectivity increase with the left angular gyrus that normalized (i.e., decreased) from postflight to follow-up (Fig. 5, Table 2).

**Table 2:** Cluster information of seed-based connectivity analyses.

Seed: contrast Labeled brain region		Peak voxel coordinates			cluster size	beta	T-value	*p*-cFWE
		x	y	z				
**Seed: Posterior cingulate cortex (postflight** > **preflight) (df** = **14)**								
No significant results								
**Seed: Posterior cingulate cortex (preflight** > **postflight) (df** = **14)**								
No significant results								
**Seed: Thalamus (sustained increases: contrast [−1 0.5 0.5]) (df** = **10)**								
No significant results								
**Seed: Thalamus (sustained decreases: contrast [1 −0.5 −0.5]) (df** = **10)**								
Superior frontal gyrus, R		24	26	40	348	0.14	7.07	<0.001
Superior frontal gyrus, L		−14	38	52	251	0.18	6.42	0.008
**Seed: Angular gyrus, R (sustained increases: contrast [−1 0.5 0.5]) (df** = **10)**								
Angular gyrus, L		−32	−64	54	178	0.17	6.78	0.044
Inferior frontal gyrus (pars triangularis), L		−32	38	04	176	0.17	6.39	0.047
**Seed: Angular gyrus, R (sustained decreases: contrast [1 −0.5 −0.5]) (df** = **10)**								
Precentral gyrus, R		40	−04	58	990	0.16	9.09	<0.001
Postcentral gyrus, L		−32	−40	64	681	0.15	7.30	<0.001
Postcentral gyrus, L		−44	−22	48	205	0.14	6.77	0.021
**Seed: bilateral insula (normalized increases: contrast [−0.5 1 −0.5]) (df** = **10)**								
Angular gyrus, L		−26	−80	42	195	0.14	5.42	0.044
**Seed: bilateral insula (normalized increases: contrast [0.5 −1 0.5]) (df** = **10)**								
Precentral gyrus (medial), R		18	−34	36	2769	0.13	9.15	<0.001
Central operculum, L		−50	02	−02	263	0.15	7.41	0.008
Central operculum, R		54	−02	18	259	0.15	6.99	0.009
Planum temporale, L		−68	−26	34	258	0.15	7.08	0.009
Supramarginal gyrus, R		48	−32	36	227	0.15	5.65	0.019

A seed-based connectivity analysis was run with the resulting clusters from the global connectivity analysis as seed regions. The contrast tested is included for each of the seeds. Beta values represent a measure for effect size; T-values are cluster-averaged; *p*-cFWE is the *p*-value after cluster-wise family-wise error (FWE) correction. This threshold was set at *p* < 0.05. Bold fonts are used as headers to define the exact test that was performed.

The complementary Bayesian analysis revealed strong evidence in favor of the alternative hypothesis for cosmonauts, confirming the pre- to postflight connectivity changes described above, while it revealed evidence for the null hypothesis in controls, indicating stable connectivity values across time for the participants who stayed on Earth (Supplementary Table 2). Two connectivity values showed anecdotal evidence for a change in controls (thalamus—right superior frontal gyrus: BF_10_ (error) = 1.02 (0.005), and right angular gyrus—left inferior frontal gyrus: BF_10_ (error) = 1.53 (9.23^−4^). However, these remained borderline values and the evidence in the cosmonaut group is much stronger (thalamus—right superior frontal gyrus: BF_10_ (error) = 548 (9.12^−6^), and right angular gyrus—left inferior frontal gyrus: BF_10_ (error) = 104 (4.86^−7^)). The interaction test revealed for all connectivity pairs evidence in favor of the alternative hypothesis, indicating that the change over time in cosmonauts is different from the change over time in controls.

### Longitudinal connectivity changes are not dependent on structural brain changes or demographic parameters

We tested for potential confounding effects of structural brain changes and demographic variability on the observed connectivity changes. In summary, we found no significant effects of age, mission duration, previous days in space, or of the interval between the time of return and the MRI scan on the connectivity changes observed in the PCC, thalamus, right angular gyrus, and the bilateral insula (Supplementary Tables 2–6, Supplementary Figs. 1–4). We also did not find correlations between gray matter or CSF volume changes within or adjacent to the clusters and connectivity changes in these same regions (Supplementary Table 7). A more detailed report on these tests is presented in the Supplementary Results.

## Discussion

Throughout evolution, Earth’s gravity has been the most stable factor, hence the human brain has evolved to function optimally under Earth’s 1 G gravity force. Nowadays, humans get exposed to prolonged microgravity and other extreme environmental factors during space missions, with still uncertain effects on brain functions^17^. Here, we aimed at studying how spaceflight influences cerebral function and evaluated how these plasticity changes re-adapt over an 8-month period after the mission. Using repeated fMRI evaluations in 15 cosmonauts, we found that functional connectivity is modulated after spaceflight and that some of these modulations are sustained over time, whereas others return to the preflight status.

Because the amount of reported data on functional connectivity changes in space crew remains scarce, we chose to perform an exploratory analysis at the whole-brain level to test whether spaceflight induced global connectivity modulations. We found that the posterior cingulate cortex (PCC) had less participation in whole-brain connectivity after spaceflight and that this reduction was sustained up to a 8-month follow-up measurement. While the role of the PCC in the brain remains quite elusive, general functions including arousal and awareness, controlling the balance of external and internal stimuli, and detecting environmental changes have been proposed^18^. From a network perspective, the PCC and precuneus belong to the default mode network (DMN)^19^, wherein they act as a central hub^20^. We found that the PCC cluster is positively correlated with typical DMN regions, including medial prefrontal cortex and bilateral angular gyri, confirming its part within the DMN. A proposed active role of the DMN is enabling long-term memory, divergent thinking patterns, and dealing with changes between world models and task sets^21^. We hypothesize that the PCC and DMN contribute to mediating adaptation triggered by unfamiliar sensory input in microgravity, based on their roles in choosing task sets, world models, and detecting environmental changes. Since the PCC is a multimodal area, it may play an overarching role in adaptation to various aspects of spaceflight. To better understand the basis of the global connectivity change, we investigated whether the PCC shows connectivity changes with specific brain regions, though we were unable to detect such effects. One possible reason is that there is a slight reduction in connectivity between the PCC and many other brain regions. Together, they would contribute to the observed global connectivity decrease, while they are not detected individually. Another explanation is that the PCC of each individual cosmonaut exhibits reduced connectivity with a unique set of brain areas, which is not revealed as significant at the group level. This would imply that the effects of spaceflight on the PCC are idiosyncratic and that each cosmonaut responds somewhat differently to the microgravity environment.

The thalamus also exhibited sustained decreased global connectivity after spaceflight. Specifically, anterior and mediodorsal thalamus were among the thalamic subregions showing altered connectivity after spaceflight. These subregions play key roles in various cognitive domains, including but not limited to working memory, spatial processing, attention, and decision making^22,23^. Our data also showed that the bilateral prefrontal cortex is coupled with this thalamus cluster and post hoc analysis revealed decreased connectivity between the thalamus and the right and left superior frontal gyri. Parnaudeau and colleagues highlight the role of the connection between mediodorsal thalamus and prefrontal cortex as an essential mediator to working memory, adaptive decision making, and assigning reward values^22^. Our results, therefore, point to changes in this tightly coupled mechanism serving these various cognitive functions.

At the same time, global connectivity increased in the right angular gyrus postflight, which sustained for 8 months after return to Earth. More specifically, this cluster encompassed both supramarginal and angular gyri. This region is known to play a part in various forms of spatial processing, including verticality assessment^24–26^. Due to the loss of gravity sensation in the ISS, cosmonauts lack their usual vertical reference and lose their sense of up and down, which similarly occurs during parabolic flight^27^. Moreover, parabolic flight was also found to induce global functional connectivity changes in the right angular gyrus^14^. Another possible explanation for the observed connectivity changes is that the right angular gyrus also contributes to action-outcome monitoring, sensory mismatch detection and sense of agency^28–30^. From these studies, the angular gyrus plays a prominent role in comparing sensory input to the expected action outcomes and thereby generating fluent motor patterns, assessing the agent of the action, and signaling mismatches. We observe from our network analysis that the right angular gyrus was positively correlated with the middle temporal gyrus, which is another key region in action-outcome predictions^30^. Microgravity can strongly violate expectations of action outcomes, while at the same time, space travelers are known to adapt to this condition over time. Therefore, we hypothesize that the unfamiliar sensory input in microgravity might drive adaptive responses in the right angular gyrus. The specific connectivity decreases between right angular gyrus and pre- and postcentral gyrus may then point to a change in the integrating role of the angular gyrus with the primary somatosensory and motor systems for generating fluent motor patterns.

Finally, testing for connectivity changes at follow-up which normalized back to preflight levels revealed a significant effect in bilateral insula. In trying to contextualize this finding, we observe that the insular cortex is linked to functions covering cognition (language, memory), perception (somesthesis, gustation), and interoception^31^. Interoceptive signals are a form of sensory input originating in the body rather than from the environment, including vestibular^32^ and visceral information^33^, and can generate unconscious autonomic responses, as well as conscious arousal responses^33,34^. In parallel, our data shows that the insular cortex is functionally connected to the salience network regions. This is a consistent finding with previous data showing the bilateral anterior insula as a key part of the salience network, together with the anterior cingulate cortex and temporo-parietal junction^35^. The function of salience networks is to select arousing stimuli and generate appropriate responses to address the relevant stimuli through attention shifts, autonomic responses, or motor responses^36^. The here identified reduced connectivity between the anterior insula and middle cingulate cortex after spaceflight points to a modification of salience network processing and may suggest a suppression of salient stimuli and autonomic responses in cosmonauts. This effect might account for a coping strategy against being exposed to conflicting and unfamiliar sensory stimuli in a microgravity environment. A well-known example of autonomic responses due to sensory conflicts in microgravity is space motion sickness, from which most of the space crew suffers during the first days in microgravity and the first days back on Earth^37^. A previous study that reports functional brain changes from another fMRI dataset in the same cosmonaut cohort found that altered connectivity between the left anterior insula and right supramarginal gyrus was correlated with space motion sickness severity^15^. Our analyses also detected connectivity changes between these two regions. Research on regular motion sickness on Earth has also revealed the involvement of salience network regions in nausea perception^38,39^ and greater autonomic response^40^ when motion sickness was provoked using vection. Moreover, the anterior insula and anterior cingulate cortex have frequently been found to activate upon vestibular stimulation during fMRI scanning^41^ and have been implicated in chronic vestibular disorders like persistent postural-perceptual dizziness^42–44^. Overall, a proposed explanation for our findings is that the processing of conflicting salient stimuli in microgravity is suppressed as an adaptation mechanism, preventing excessive arousal, and resulting autonomic responses in microgravity.

Our results show parallels with related work on functional brain reorganization after simulated spaceflight by means of the head-down bedrest (HDBR) model. One study showed altered brain activity in the mediodorsal thalamus after only 72 h of HDBR^45^ and another study reported connectivity changes in the inferior parietal lobule, which correlated with performance on a mental transformation task^46^. Seven days of HDBR resulted in regional homogeneity decreases in posterior cingulate cortex and increases in anterior cingulate cortex^47^. Zhou and colleagues found that the anterior insula and dorsal anterior cingulate cortex showed altered network centrality and functional connectivity, pertaining to the key salience network regions similar to our findings^48^. Longer periods of HDBR result in connectivity changes that are rather related to sensorimotor and vestibular functioning^49–52^. Lastly, the role of the DMN in microgravity-dependent adaptation has been researched in various ways. In one study, the authors used a classifying algorithm to predict the gravity state based on DMN connectivity^53^. In another series of studies, heart rate variability was used to indirectly assess brain function before, during, and after spaceflight^54,55^, based on the notion that cortical brain function, and specifically the DMN, influences the heart through the autonomic nervous system^56^. Overall, having matching results across studies adopting both actual and simulated spaceflight increasingly supports such regions’ involvement in microgravity-induced adaptation.

In our study, we demonstrate that connectivity changes due to long-duration spaceflight can both sustain or reverse to preflight levels when taking measurements over a half year after return to Earth. It is somewhat surprising that connectivity changes in PCC, thalamus, and right angular gyrus sustain for at least 8 months after the space mission, given that space travelers functionally re-adapt to Earth’s environment in days to weeks. Nevertheless, adaptive plasticity can range from temporary compensatory reorganization to permanent reorganization in the brain^57^. Given that certain professional groups can present with distinguished neural characteristics compared to the general population (see e.g., refs. ^58–60^.), there are reasons to believe permanent learning behavior and associated neural organization may occur following extensive training of sensorimotor functions in microgravity. However, the possibility remains that these effects can eventually reverse over a longer term after the space mission, for which we cannot provide the data yet. Besides sustained effects, our study additionally shows that the connectivity alterations of the insular cortex and salience network are normalized to baseline levels 7 months after cosmonauts returned to Earth. Such reversibility suggests that salience processing in the brain adapts according to the gravitational condition, i.e., suppressed in microgravity or enhanced in Earth’s 1 G. An alternative explanation is that salience network modulation is a transient phenomenon, which occurs in response to a gravitational transition. The latter explanation would be more in line with observations regarding space motion sickness, which occurs during the first days in microgravity and the first days back on Earth.

Functional connectivity changes generally indicate altered communication patterns within the brain. We interpret these findings as adaptation mechanisms to the microgravity environment, though several alternative explanations and confounding factors are to be considered as well. First, it is known that structural brain changes occur in space crew, which mainly point to tissue remodeling and fluid redistributions. To date, it is unclear what its functional or behavioral consequences are, although some studies have shown functional impairments related to brain structural changes^7,61,62^. We investigated whether functional connectivity changes correlated with gray matter volume changes in the PCC, thalamus, right angular gyrus, and insular cortex, and we found no significant correlation. The structural basis for our functional connectivity findings may therefore originate rather at the synaptic level^63,64^. In this framework, we hypothesize that functional connectivity changes could result from altered ion channel expressions, spine formations, and dendritic branching, which are cellular and molecular hallmarks of neuroplasticity. Nevertheless, the possibility that the gray matter volume changes lead to the observed functional connectivity changes cannot be fully ruled out. Second, functional connectivity measures have a vascular basis and cerebral blood perfusion has been shown to be affected by HDBR^65^, although no study has demonstrated this yet in space crew. Third, functional connectivity changes may represent neural correlates for impaired performance and central nervous system function, rather than beneficial adaptation. Moreover, various other space stressors besides microgravity, such as sleep deprivation, isolation, confined living quarters, and high workloads may impact brain functional architecture.

Limitations of the current study are due to several logistic difficulties related to spaceflight studies. First, our postflight data is acquired on average nine days after Earth re-entry, allowing for changes that occurred during these first nine days to be picked up by our data. Similarly, the preflight scan is sometimes scheduled longer before launch than anticipated, which eventually led to a nearly significant difference in scan time interval between the cosmonaut and control groups in our data. Second, the cosmonaut sample is small and therefore might have lowered the statistical power for evidencing more nuanced results. This drawback is a natural consequence of the fact that only a few humans travel to space, and dropouts can also happen across the multiple scanning sessions. Also, most space travelers go on multiple missions during their career, which inevitably results in acquiring data in a mix of first-time flyers and experienced flyers. Lastly, we were unable to acquire behavioral data in our study to link neuroimaging results with individual cosmonaut performance, due to, among many logistic difficulties, time constraints for access to cosmonaut testing. Moreover, the reported brain regions in this work are multimodal in nature and there remains an incomplete knowledge on the full scope of these regions’ functions in the brain in general, let alone their potential role in adapting to the unique conditions of spaceflight. Establishing an integrated scheme of structural and functional changes that occur after spaceflight in more detail will be crucial to allow for developing potential countermeasures, to monitor proper brain function during space missions, and to better understand how longer duration space missions influence these structural and functional changes. Future work can make use of the brain regions shown to exhibit connectivity changes in this study to conduct more directed neuroimaging analyses. Alternatively, behavioral and functional task experiments for astronauts that are associated with these regions’ roles in the brain can be adopted to better understand the functional implications of the current findings, both for spaceflight and spaceflight analog studies.

## Methods

### Participants

Thirteen male Russian cosmonauts, engaged in long-duration space missions to the ISS between February 2014 and February 2020, gave their consent to participate in the current study. Data were acquired prospectively before their mission (preflight), shortly after (postflight), and ~8 months later (follow-up). Two cosmonauts enrolled in the study twice for two consecutive spaceflight missions and fully concluded the preflight, postflight, and follow-up scanning sessions both times. Follow-up data for four cosmonauts are missing in this dataset. This leads to a total of 15 preflight and postflight scans, and 11 scans acquired at the follow-up assessment. Fourteen healthy participants matched for age, gender, education, and handedness were included as controls to assess spontaneous connectivity effects due to time. The control group was scanned at two timepoints, with an interval similar to that of the cosmonauts’ preflight and postflight scans. All cosmonauts and controls were right-handed. Details on the demographics of both groups are summarized in Table 3. The age and the scan interval were statistically compared between cosmonauts and controls by means of a two-tailed Mann–Whitney U test and an exact significance threshold of *p* < 0.05. The study was approved by the Institutional Review Board of the Antwerp University Hospital (13/38/357), the European Space Agency Medical Board, the Committee of Biomedicine Ethics of the Institute of Biomedical Problems of the Russian Academy of Science, and the Human Research Multilateral Review Board. All participants provided a signed informed consent, and all investigations were performed in accordance with the principles listed in the Declaration of Helsinki and its amendments.

**Table 3:** Demographic information of the cosmonaut and control groups (all males).

	Cosmonauts median (MAD)	Controls median (MAD)	Mann–Whitney U test(*p*-value)
Age (years)	45.0 (2.4)	41 (4)	0.134
Mission duration (days)	173 (9)		
Previous mission experience (days)	165 (32)		
Preflight MRI—launch interval (days)	96 (26)		
Re-entry—postflight MRI interval (days)	9 (1)		
Preflight MRI—postflight MRI interval (days)	284 (18)	221 (38)	0.051
Re-entry—follow-up MRI interval (days)	239 (45)		

The last column represents the results of the Mann–Whitney U test comparing age and scan intervals between cosmonauts and controls.

*MAD* median absolute deviation.

### MRI acquisition

Resting-state fMRI data were acquired on a 3 T MRI scanner (Discovery MR750; GE Healthcare USA) located at the Federal Center of Treatment and Rehabilitation in Moscow, Russia. T2* weighted echo planar imaging scans were acquired using a 16-channel head and neck array coil with the participants positioned head-first and supine. The following scanning parameters were used: echo time = 30 ms, repetition time = 2000ms, flip angle = 77°, voxel size = 3 × 3 × 3 mm³, field of view = 192 × 192 × 126 mm (matrix dimension: 64 × 64, 42 axial slices). A total of 300 images per session were acquired after 4 dummy scans (8 s) to achieve steady-state conditions. Participants were instructed to keep their eyes closed, to let their mind wander and not think of anything in particular, while not falling asleep. In addition, a high-resolution fast-spoiled gradient echo (FSPGR) 3D T1-weighted image was acquired for the purpose of anatomical localization. The scanning parameters included: echo time = 3.06 ms, repetition time = 7.90 ms, inversion time = 450 ms, flip angle = 12°, voxel size = 1 mm³, field of view = 176 × 240 × 240 mm (matrix dimensions: 240 × 240, 176 sagittal slices).

### Data preprocessing

Preprocessing was performed for each participant using SPM12 (revision 7219; Wellcome Department of Cognitive Neurology, London, UK) run on Matlab R2018b (Mathworks, Natick, MA, USA) as follows: Structural and functional images were first manually reoriented to the Montreal Neurological Institute (MNI) template to assure rough spatial correspondence. Functional MRI images then underwent slice-time correction, realignment to the first volume, and coregistration to the T1-weighted structural images. The T1 images were segmented into gray matter, white matter and CSF and were bias field corrected. Afterwards, functional and structural images were non-linearly warped and normalized to MNI space for a final spatial resolution of 3 × 3 × 3 mm for the functional and 1 × 1 × 1 mm for the anatomical images. Functional images were eventually smoothed with a Gaussian kernel of 6 × 6 × 6 mm³ at full-width half-maximum. For noise reduction, the anatomical component-based noise correction aCompCor method was used^66^. This approach models the influence of noise as a voxel-specific linear combination of multiple empirically estimated noise sources by deriving principal components from noise regions of interest (ROI) and by including them as nuisance parameters within the first-level general linear model. Specifically, the segmented white matter and CSF masks were eroded by one voxel to minimize partial volume effects with the gray matter masks, which resulted in substantially smaller masks than the original segmentations. The eroded white matter and CSF masks were then used as noise ROIs. Signals from the white matter and CSF noise ROIs were extracted from the unsmoothed functional volumes to avoid additional risk of contaminating the gray matter signal with white matter and CSF signals. A temporal band-pass filter of 0.008–0.09 Hz and linear detrending was applied. Residual head motion parameters (three rotation and three translation parameters, plus another six parameters representing their first-order temporal derivatives) were entered as noise covariates. Additional motion correction was performed by estimating outlier images which were entered as extra regressors of no interest in the design matrix. Motion outliers were defined by means of the artifact detection toolbox (ART; https://www.nitrc.org/projects/artifact_detect/) using a global signal threshold of z = 3.0, an absolute motion threshold of 0.5 for translation and 0.05 for rotation, and a scan-to-scan motion threshold of 1.0 for translation and 0.02 for rotation.

### Connectivity analysis

The analysis embraced a repeated-measures design. Connectivity analysis was performed using the functional connectivity toolbox (CONN v18; https://www.nitrc.org/projects/conn). A hypothesis-free voxel-to-voxel analysis was carried out to investigate functional connectivity changes within a whole-brain mask using the Intrinsic Connectivity Contrast (ICC). The ICC is a measure based on network centrality (degree). At a given reference voxel *i*, there is a connectivity value (i.e. correlation coefficient) between this voxel and voxel *j*, expressed as r(i, j). For the ICC to be computed, the connectivity values between voxel *i* and every other voxel are weighted by their r^2^ value and averaged. For statistical purposes, we chose to normalize these values to fit a Gaussian distribution with zero mean and unitary variance, by subtracting the ICC value obtained at each voxel by the average value across all the voxels and dividing this by the standard deviation of the whole-brain map. Therefore, the ICC represents a global connectivity value of a given voxel and all the other voxels, without the need for a correlation threshold, hence providing a metric that requires no a priori information either in terms of arbitrary thresholds or ROI definitions. Consequently, the ICC considers not only the presence of a connection but also the strength of these connections^67^.

### Statistics and reproducibility

All voxel-based statistics were performed through CONN v18. At the first-level, the ICC was defined for each subject and condition separately, estimating the resulting beta-maps from the voxel-to-voxel connectivity matrix for each subject and for each scanning condition (preflight, postflight, follow-up). At the second-level (group level), the contrasts of interest were defined, and the second-level results were estimated. Initially, we compared pre- and postflight data in controls as a sanity check, expecting no change in connectivity across time. Afterwards, the pre- and postflight data in cosmonauts were compared to identify changes in connectivity, expecting effects of spaceflight on ICC. Both comparisons were run using a two-tailed paired *t*-test. A threshold of *p* < 0.005 uncorrected at the voxel level was applied, followed by a threshold of *p* < 0.05 at the cluster level and corrected using family-wise error (FWE).

Next, Bayesian statistics were used to verify the effects in each cosmonaut-identified region of connectivity change (clusters): the average connectivity values of each cluster were extracted for each time point and for each cosmonaut and control participant. The Bayes Factor (BF) was estimated in favor of the alternative hypothesis H_1_ over the null hypothesis H_0_ (BF_10_) for the comparison of pre- and postflight data in cosmonauts, for the comparison between the two measurements in controls, and finally for the comparison of cosmonauts and controls regarding the difference in connectivity between the two scans over time. All analyses used a Cauchy prior distribution with a default width of 0.707. BF_10_ values between 0 and 1 correspond to evidence in favor of H_0_, while values above 1 correspond to evidence in favor of H_1_. The degree of evidence for H_1_ is categorized into anecdotal (values 1–3), moderate (values 3–10), strong (values 10–30), very strong (values 30–100), and extreme (values > 100), with similar categories for the inverse values defining evidence for H_0_, as implemented in JASP (JASP Team 2019, version 0.11.1).

For those cosmonauts (*n* = 11) scanned at three timepoints (preflight, postflight, follow-up), longitudinal connectivity effects were tested using two-tailed repeated-measures T-contrasts: for the sustained effects, the sustain model was used for identifying postflight increases [−1 0.5 0.5] and decreases [1 −0.5 −0.5] which remained at follow-up. For the normalized effects, the normalize model was used to identify postflight increases [−0.5 1 −0.5] and decreases [0.5 −1 0.5] which returned to the preflight baseline at follow-up. Statistical parametric maps for all analyses were evaluated at a whole-brain voxel-level threshold of *p* < 0.005 uncorrected, and a cluster-wise inference adopting FWE correction for multiple comparisons at *p* < 0.05.

The functional networks associated with the resulting clusters from the ICC analysis were estimated to confirm the clusters’ roles in well-defined resting-state networks based on the work by Yeo et al.^68^ and to aid in results interpretation. First, the identified ICC clusters were used as binary masks serving as seed ROIs in classical connectivity investigation. For each participant, the average timeseries within each of these seed ROIs were calculated and then correlated with the timeseries extracted within each voxel in the brain. The obtained r correlation values were then transformed into Gaussian values by means of the Fisher transform (z-scores). A one-sample *t*-test was ordered to test which z-scores are significantly different from zero combining data from all sessions in cosmonauts. A statistical voxel-level threshold of *p* < 0.001 uncorrected at the whole-brain level was applied, followed by a cluster-level threshold of *p* < 0.05 FWE-corrected. Resulting clusters were overlaid on an atlas containing seven resting-state networks^68^. Next, the longitudinal connectivity changes between the seed ROI and all other voxels were calculated with the above-mentioned sustain and normalize models. To minimize the number of comparisons, only one test was performed for each seed ROI, namely the test that led to the significant ICC changes in the first place (e.g., if the sustain model showed a significant ICC change in a cluster, only the sustain model was used with that cluster as seed ROI in the classical connectivity analysis). For all tests, statistical thresholding was applied using an uncorrected whole-brain voxel-level threshold at *p* < 0.005, with an additional cluster level *p* < 0.05 FWE correction for multiple comparisons.

Correlation analyses were performed to associate connectivity changes with demographic information of the cosmonauts, with the timing of MRI acquisition, and with structural changes within these same regions. For a detailed description of these methods, the reader is referred to the Supplementary Methods.

MRI data visualizations regarding background-overlay images were done with the MRI viewer of MRtrix3^69^, semi-inflated 3D brain maps were created in CONN, and brain labeling was done using the Neuromorphometrics atlas (http://www.oasis-brains.org/; http://Neuromorphometrics.com/).

### Reporting summary

Further information on research design is available in the Nature Portfolio Reporting Summary linked to this article.

## Supplementary information

Supplementary Information

Description of Additional Supplementary Files

Supplementary Data 1

Reporting summary

## Data Availability

The whole-brain T-score maps for all statistical tests that were run have been uploaded to Neurovault (https://neurovault.org/collections/12152/). All extracted connectivity values from each cluster from the ICC analysis are provided as supplementary data in xlsx format. Per request, other data files may be provided as well by contacting steven.jillings@uantwerpen.be.

## Code availability

Custom codes were used neither for data acquisition nor for data analyses that were central to reaching the conclusions of this work.
