# Supplementary material for: Prolonged microgravity induces reversible and persistent changes on human cerebral connectivity
Source: Commun Biol. 2023 Jan 13;6:46. doi: 10.1038/s42003-022-04382-w (PMC9839680; doi:10.1038/s42003-022-04382-w)
Supplement: Supplementary file 1 — Supplementary Information [file 42003_2022_4382_MOESM1_ESM.pdf]

Supplementary material to "prolonged microgravity induces reversible and persistent changes on human cerebral connectivity"

Content List

1) Supplementary Methods

2) Supplementary Results

3) Supplementary Figures and Tables

---

1) Supplementary Methods

To test whether interindividual variation in longitudinal connectivity changes in cosmonauts can be explained by demographic information, we performed linear mixed model (LMM) analyses. Connectivity values were obtained in the posterior cingulate cortex (PCC), thalamus, right angular gyrus (rAG), and bilateral insula from the *sustain* and *normalize* contrasts adopted in the main manuscript. Specifically, the intrinsic connectivity values were averaged across the voxels for each cluster separately and for each time point separately. Time, age, mission duration, previous days in space, and each of the interaction effects with time were included as fixed effects, with time being a categorical variable. Subject was considered as a random effect and a random intercept model was adopted. For the first LMM analysis, we included only the pre- and post-flight data and added the interval between the return date and the post-flight MRI scan (*postflight MRI delay)*, as well as its interaction with time, to the list of fixed effects. For the second LMM analysis, we included only the preflight and follow-up data and added the interval between the return date and the follow-up MRI scan (*follow-up MRI delay)*, as well as its interaction with time, as fixed effects. We particularly aimed to test whether the interaction terms were significant variables in the model as a means to evaluate each variable's contribution in explaining connectivity changes in time. A threshold of $p<0.05$ was applied to determine statistical significance. Through an iterative process, the least significant interaction term, along with the single variable, is discarded and the model is re-run until only significant interaction terms remain.

Next, we performed correlation analyses of connectivity changes with structural changes in the PCC, thalamus, rAG, and insula. Specifically, we correlated each region's connectivity changes to the grey matter (GM) volume changes from pre- to post-flight. Additionally, correlations were tested between thalamic connectivity changes and the adjacent third ventricular volume changes, as well as between the bilateral insular connectivity changes and the cerebrospinal fluid (CSF) volume change within that cluster. GM and CSF volumes within the PCC, thalamus, rAG, and insula clusters were estimated using the *Tissue Volumes* utility

in SPM12 (v7771) by providing the GM segmentation outputs resulting from the T1w image preprocessing steps (see main manuscript) and by specifying the clusters where connectivity changes were observed as masks. The third ventricular volume was estimated using the standard segmentation and normalisation algorithms of CAT12 (r1932) and using the Neuromorphometrics atlas (http://www.oasis-brains.org/; http://Neuromorphometrics.com/). A threshold of $p<0.05$ was applied to determine statistical significance.

2) Supplementary Results

The linear mixed models revealed significant effects of time on the pre- and post-flight connectivity data of PCC (p=0.0008), thalamus (p=0.0001), rAG (p=0.0007) and insula (p=0.0005). None of the interaction terms between time on the one hand and age, mission duration, previous days in space, and the post-flight MRI delay on the other hand showed significant effects for the connectivity data in PCC, thalamus, rAG or insula (**tables S3-S6**).

Likewise, time had a significant effect on the preflight and follow-up connectivity data of PCC (p=0.004), thalamus (p=0.0003), and rAG (p=0.010), while it did not have a significant effect for the bilateral insula, reflecting the normalised connectivity changes as measured at follow-up. Again, there were no significant effects of the interaction between time on the one hand and age, mission duration, previous days in space, and the follow-up MRI delay on the other hand on the preflight and follow-up connectivity data (**tables S3-S6**).

There were also no significant correlations between connectivity changes and GM volume in any of the tested regions (**table S7**). Additionally, there was no significant correlation between thalamic connectivity changes and ventricular volume change, nor between connectivity changes and CSF volume changes in the insular cortex.

Altogether, these findings show that the tested demographic variables and brain structural changes due to spaceflight do not confidently explain the variance observed in the connectivity changes in this space crew cohort. Graphical illustrations of the tested correlations are presented in **Figures S1-S4.**

3) Supplementary Figures and Tables

		Cosmonauts		Controls		Cosmo vs Controls	
**Brain region**	**direction**	**BF_{10} (error)**	**Evidence for H1**	**BF_{10} (error)**	**Evidence for H0**	**BF10 (error)**	**Evidence for H1**
Intrinsic connectivity contrast (ICC) analysis							
PCC, L	sus -	51 (6^{-5})	VS	0.270 (8^{-3})	M	7 (5^{-5})	M
Thalamus, L	sus -	245 (2^{-5})	E	0.271 (8^{-3})	M	7 (5^{-5})	M
Angular gyrus, R	sus +	53 (7^{-5})	VS	0.367 (1^{-2})	A	33 (3^{-5})	VS
Insular cortex, BL	rev -	69 (5^{-5})	VS	0.270 (8^{-3})	M	19 (2^{-4})	S

Table S1. Bayesian statistical analyses for the significant clusters of the global connectivity analysis in cosmonauts. The evidence for an effect of time in each of the clusters is given for cosmonauts as a reference and for controls to show whether there was an effect of time in this group. BF_{10} is the Bayesian factor in favor of the alternative hypothesis (i.e. there is an effect of time). Values >1 correspond to evidence for the alternative hypothesis, while values <1 correspond to evidence for the null hypothesis (i.e. no difference in time). The strength of the evidence is given by anecdotal (A), moderate (M), strong (S), very strong (VS) and extreme (E) according to the convention used in the statistical program JASP.

		Cosmonauts		Controls		Cosmo vs Controls	
**Brain region**	**+/-**	**BF_{10} (error)**	**evidence for H1**	**BF_{10} (error)**	**evidence for H0**	**BF10 (error)**	**evidence for H1**
**Seed ROI: Thalamus (sustained effects)**							
superior frontal gyrus, R	-	548 (9e-6)	E	1 (5e-3)	A*	2 (1e-3)	A
superior frontal gyrus, R	-	255 (1e-5)	E	0.5 (2e-2)	A	2 (1e-3)	A
**Seed ROI: right angular gyrus (sustained effects)**							
precentral gyrus, R	-	147 (3e-5)	E	0.3 (8e-3)	M	8 (1e-4)	M
postcentral gyrus, L	-	43 (5e-4)	VS	0.3 (9e-3)	M	7 (5e-5)	M
postcentral gyrus, L	-	5 (1e-4)	M	0.3 (1e-2)	A	2 (3e-3)	A
angular gyrus, L	+	154 (4e-5)	E	0.3 (9e-3)	M	15 (3e-4)	S
inferior frontal gyrus, L	+	104 (5e-7)	E	2 (9e-4)	A*	537 (3e-6)	E
**Seed ROI: bilateral insular cortex (normalized effects)**							
precentral gyrus, R	-	76 (4e-5)	VS	0.4 (1e-2)	A	10 (2e-4)	S
central operculum, L	-	13 (6e-5)	S	0.3 (9e-3)	M	4 (5e-5)	M
central operculum, R	-	170 (4e-5)	E	0.4 (1e-2)	A	41 (9e-5)	VS
planum temporale, L	-	15 (6e-5)	S	0.5 (2e-2)	A	1 (2e-3)	A
supramarginal gyrus, R	-	42 (4e-5)	VS	0.5 (2e-2)	A	16 (2e-4)	S
angular gyrus, L	+	32 (7e-6)	VS	0.3 (1e-2)	A	13 (3e-4)	S

Table S2. Bayesian statistical analyses for the significant clusters of the seed-to-voxel analysis in cosmonauts. The evidence for an effect of time in each of the clusters is given for cosmonauts, for controls, and for the difference between the two groups (interaction effect group*time). BF_{10} is the Bayesian factor in favour of the alternative hypothesis (i.e. there is an effect of time). Values >1 correspond to evidence for the alternative hypothesis, while values <1 correspond to evidence for the null hypothesis (i.e. no difference in time). The strength of the evidence is given by anecdotal (A), moderate (M), strong (S), very strong (VS) and extreme (E) according to the convention used in the statistical program JASP. The second columns indicates the direction of connectivity change from pre- to postflight by (+) for an increase and (-) for a decrease. *In these cases, anecdotal evidence in favour of H1 instead of H0.

Posterior cingulate cortex (PCC)										
**Post vs. Pre**	**All variables included**					**After removing non-significant variables**				
VAR	Est	S.E.	DF	t	p	Est	S.E.	DF	t	p
Intercept	1.7	2.3	8.03	0.77	0.46	0.66	0.14	15.48	4.72	0.0003
Time	0.64	0.11	6.00	5.63	**0.0013**	0.64	0.13	10.00	4.76	**0.0008**
Age	-0.01	0.06	8.03	-0.09	0.93	-	-	-	-	-
Age*Time	-0.11	0.04	6.00	-2.34	0.06	-	-	-	-	-
MD	-0.001	0.005	8.03	-0.26	0.80	-	-	-	-	-
MD*Time	0.006	0.004	6.00	1.75	0.13	-	-	-	-	-
PDS	0.0002	0.0013	8.03	0.13	0.90	-	-	-	-	-
PDS*Time	0.002	0.001	6.00	1.79	0.12	-	-	-	-	-
Interval	-0.07	0.06	8.03	-1.09	0.31	-	-	-	-	-
Interval*Time	0.03	0.04	6.00	0.60	0.57	-	-	-	-	-
**Fol vs. Pre**	**All variables included**					**After removing non-significant variables**				
VAR	Est	S.E.	DF	t	p	Est	S.E.	DF	t	p
Intercept	0.1	1.7	11.88	0.07	0.94	0.72	0.13	18.98	5.66	<0.0001
Time	0.58	0.14	6.00	4.07	**0.007**	0.58	0.16	10.00	3.72	**0.0040**
Age	-0.00	0.05	11.88	-0.06	0.96	-	-	-	-	-
Age*Time	-0.12	0.06	6.00	-1.86	0.11	-	-	-	-	-
MD	-0.002	0.004	11.88	-0.63	0.54	-	-	-	-	-
MD*Time	0.008	0.005	6.00	1.69	0.14	-	-	-	-	-
PDS	0.0007	0.0011	11.88	0.58	0.57	-	-	-	-	-
PDS*Time	0.0020	0.0015	6.00	1.30	0.24	-	-	-	-	-
Interval	0.0043	0.0025	11.88	1.73	0.11	-	-	-	-	-
Interval*Time	-0.006	0.003	6.00	-1.95	0.10	-	-	-	-	-

Table S3. Model parameters for the influence of demographics on connectivity data of the posterior cingulate cortex (PCC). The model included subject as random effect and time, age, mission duration (MD), previous days in space (PDS), and the interval between the return date and the post-flight (post) MRI scan (top half) or follow-up (fol) MRI scan (bottom half) as fixed effects. Non-significant variables were subsequently removed from the model and the analyses was re-run to obtain the final model parameters (right half). Est=estimate, S.E.=standard error on the estimate, DF=degrees of freedom, t=t-statistic, p=p-value.

Thalamus										
**Post vs. Pre**	**All variables included**					**After removing non-significant variables**				
VAR	Est	S.E.	DF	t	p	Est	S.E.	DF	t	p
Intercept	1.1	1.4	8.81	0.78	0.46	-0.50	0.11	12.75	-4.55	0.0006
Time	0.47	0.08	6.00	5.80	**0.001**	0.47	0.08	10.00	6.07	**0.0001**
Age	-0.02	0.04	8.81	-0.50	0.63	-	-	-	-	-
Age*Time	0.05	0.03	6.00	1.40	0.21	-	-	-	-	-
MD	-0.003	0.003	8.81	-1.00	0.34	-	-	-	-	-
MD*Time	-0.003	0.003	6.00	-1.28	0.25	-	-	-	-	-
PDS	0.0008	0.0008	8.81	0.96	0.36	-	-	-	-	-
PDS*Time	-0.0012	0.0007	6.00	-1.70	0.14	-	-	-	-	-
Interval	-0.04	0.04	8.81	-1.02	0.33	-	-	-	-	-
Interval*Time	-0.01	0.03	6.00	-0.36	0.73	-	-	-	-	-
**Fol vs. Pre**	**All variables included**					**After removing non-significant variables**				
VAR	Est	S.E.	DF	t	p	Est	S.E.	DF	t	p
Intercept	-2.3	1.6	9.42	-1.42	0.19	0.52	0.12	13.00	-4.23	0.0010
Time	0.49	0.10	6.00	5.02	**0.002**	0.49	0.09	10.00	5.43	**0.0003**
Age	0.07	0.04	9.42	1.68	0.13	-	-	-	-	-
Age*Time	-0.03	0.04	6.00	-0.88	0.41	-	-	-	-	-
MD	-0.008	0.003	9.42	-2.37	**0.04**	-	-	-	-	-
MD*Time	0.002	0.003	6.00	0.63	0.55	-	-	-	-	-
PDS	-0.0002	0.0011	9.42	-0.17	0.87	-	-	-	-	-
PDS*Time	0.0001	0.0010	6.00	0.08	0.94	-	-	-	-	-
Interval	0.0003	0.0023	9.42	0.11	0.92	-	-	-	-	-
Interval*Time	-0.0011	0.0023	6.00	-0.49	0.64	-	-	-	-	-

Table S4. Model parameters for the influence of demographics on connectivity data of the thalamus The model included subject as random effect and time, age, mission duration (MD), previous days in space (PDS), and the interval between the return date and the post-flight (post) MRI scan (top half) or follow-up (fol) MRI scan (bottom half) as fixed effects. Non-significant variables were subsequently removed from the model and the analyses was re-run to obtain the final model parameters (right half). Est=estimate, S.E.=standard error on the estimate, DF=degrees of freedom, t=t-statistic, p=p-value.

Right Angular Gyrus										
**Post vs. Pre**	**All variables included**					**After removing non-significant variables**				
VAR	Est	S.E.	DF	t	p	Est	S.E.	DF	t	p
Intercept	-0.7	1.5	9.83	-0.45	0.67	1.39	0.11	19.28	12.66	<0.0001
Time	-0.81	0.16	6.00	-4.94	**0.003**	-0.81	0.17	10.00	-4.79	**0.0007**
Age	0.09	0.04	9.83	2.41	**0.037**	-	-	-	-	-
Age*Time	-0.07	0.07	6.00	-1.09	0.32	-	-	-	-	-
MD	-0.009	0.003	9.83	-3.13	**0.01**	-	-	-	-	-
MD*Time	0.009	0.005	6.00	1.74	0.13	-	-	-	-	-
PDS	-0.0024	0.0008	9.83	-2.86	**0.017**	-	-	-	-	-
PDS*Time	0.0026	0.0014	6.00	1.78	0.13	-	-	-	-	-
Interval	0.01	0.04	9.83	0.32	0.76	-	-	-	-	-
Interval*Time	0.05	0.07	6.00	0.70	0.51	-	-	-	-	-
**Fol vs. Pre**	**All variables included**					**After removing non-significant variables**				
VAR	Est	S.E.	DF	t	p	Est	S.E.	DF	t	p
Intercept	-2.3	2.1	9.27	-1.08	0.31	0.95	0.13	14.82	7.33	<0.0001
Time	-0.37	0.12	6.00	-2.97	**0.03**	-0.37	0.12	10.00	-3.17	**0.010**
Age	0.05	0.06	9.27	0.89	0.40	-	-	-	-	-
Age*Time	-0.02	0.05	6.00	-0.39	0.71	-	-	-	-	-
MD	-0.003	0.004	9.27	-0.65	0.53	-	-	-	-	-
MD*Time	0.001	0.004	6.00	0.26	0.80	-	-	-	-	-
PDS	-0.0014	0.0014	9.27	-1.02	0.33	-	-	-	-	-
PDS*Time	0.0006	0.0013	6.00	0.49	0.64	-	-	-	-	-
Interval	0.008	0.003	9.27	2.52	**0.03**	-	-	-	-	-
Interval*Time	-0.0046	0.0029	6.00	-1.58	0.16	-	-	-	-	-

Table S5. Model parameters for the influence of demographics on connectivity data of the right angular gyrus. The model included subject as random effect and time, age, mission duration (MD), previous days in space (PDS), and the interval between the return date and the post-flight (post) MRI scan (top half) or follow-up (fol) MRI scan (bottom half) as fixed effects. Non-significant variables were subsequently removed from the model and the analyses was re-run to obtain the final model parameters (right half). Est=estimate, S.E.=standard error on the estimate, DF=degrees of freedom, t=t-statistic, p=p-value.

Bilateral Insula										
**Post vs. Pre**	**All variables included**					**After removing non-significant variables**				
VAR	Est	S.E.	DF	t	p	Est	S.E.	DF	t	p
Intercept	-0.1	1.3	11.88	-0.05	0.96	-0.19	0.09	19.97	-2.23	0.038
Time	0.60	0.12	6.00	5.15	**0.002**	0.60	0.12	10.00	5.01	**0.0005**
Age	0.00	-0.04	11.88	-0.12	0.91	-	-	-	-	-
Age*Time	-0.03	0.05	6.00	-0.60	0.57	-	-	-	-	-
MD	-0.0004	0.0027	11.88	-0.15	0.88	-	-	-	-	-
MD*Time	0.000	0.004	6.00	0.10	0.93	-	-	-	-	-
PDS	0.0004	0.0008	11.88	0.48	0.64	-	-	-	-	-
PDS*Time	-0.0006	0.0010	6.00	-0.58	0.58	-	-	-	-	-
Interval	0.01	0.04	11.88	0.20	0.84	-	-	-	-	-
Interval*Time	-0.10	0.05	6.00	-2.09	0.08	-	-	-	-	-
**Fol vs. Pre**	**All variables included**					**After removing non-significant variables**				
VAR	Est	S.E.	DF	t	p	Est	S.E.	DF	t	p
Intercept	3.1	1.9	9.63	1.59	0.14	-	-	-	-	-
Time	-0.02	0.21	6.00	-0.10	0.92	-	-	-	-	-
Age	-0.06	0.05	9.63	-1.25	0.24	-	-	-	-	-
Age*Time	0.06	0.09	6.00	0.66	0.53	-	-	-	-	-
MD	0.008	0.004	9.63	1.88	0.09	-	-	-	-	-
MD*Time	-0.008	0.007	6.00	-1.19	0.28	-	-	-	-	-
PDS	0.0004	0.0012	9.63	0.32	0.75	-	-	-	-	-
PDS*Time	-0.0003	0.0021	6.00	-0.14	0.89	-	-	-	-	-
Interval	-0.0053	0.0028	9.63	-1.92	0.08	-	-	-	-	-
Interval*Time	0.005	0.005	6.00	1.05	0.33	-	-	-	-	-

Table S6. Model parameters for the influence of demographics on connectivity data of the bilateral insula. The model included subject as random effect and time, age, mission duration (MD), previous days in space (PDS), and the interval between the return date and the post-flight (post) MRI scan (top half) or follow-up (fol) MRI scan (bottom half) as fixed effects. Non-significant variables were subsequently removed from the model and the analyses was re-run to obtain the final model parameters (right half). Est=estimate, S.E.=standard error on the estimate, DF=degrees of freedom, t=t-statistic, p=p-value.

Comparison	pearson's r	p-value
PCC ∆FC vs. ∆GMV	-0.264	0.432
Thalamus ∆FC vs. ∆GMV	-0.352	0.289
Thalamus ∆FC vs. ∆ventricular CSF volume	0.002	0.995
rAG ∆FC vs. ∆GMV	-0.304	0.363
Bilateral Insula ∆FC vs. ∆GMV	0.277	0.409
Bilateral Insula ∆FC vs. ∆CSF volume	-0.196	0.564

Table S7. Correlation analyses between structural and functional changes. Pre- to post-flight changes in functional connectivity (∆FC) and changes in grey matter volume (∆GMV) or cerebrospinal fluid (CSF) volume were correlated in the posterior cingulate cortex (PCC), thalamus, right angular gyrus (rAG), and bilateral insula.

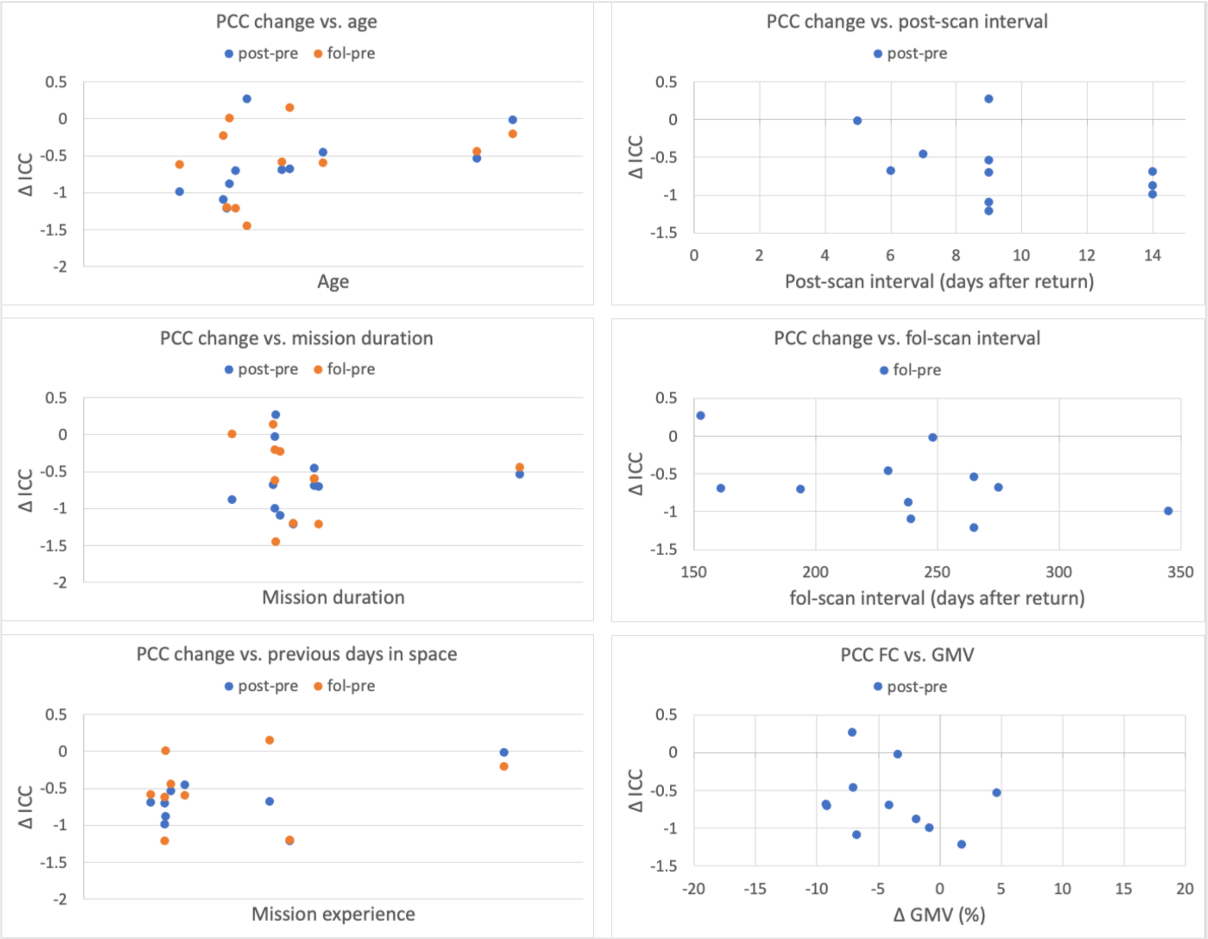

Figure S1. Connectivity change of the posterior cingulate cortex (PCC) in function of demographic variables and grey matter volume changes. Scatterplots of the connectivity (ICC) changes from pre- to post-flight (blue) and preflight to follow-up (orange) with age, mission duration, mission experience, interval between return date and postflight MRI, interval between return date and follow-up MRI, and pre- to postflight change in grey matter volume (ΔGMV). Values for age, mission duration and mission experience are excluded to avoid subject identification.

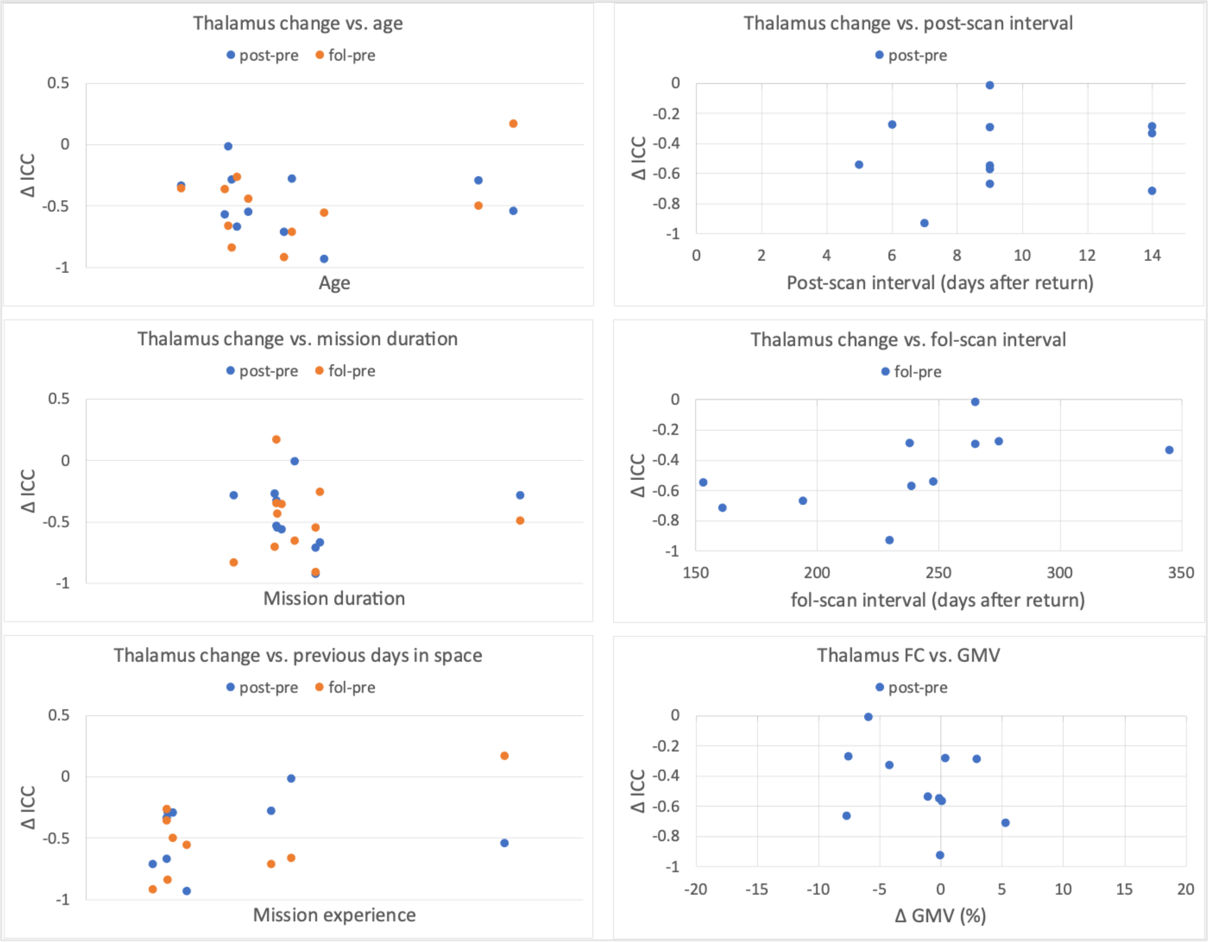

Figure S2. Connectivity change of the thalamus in function of demographic variables and grey matter volume changes. Scatterplots of the connectivity (ICC) changes from pre- to post-flight (blue) and preflight to follow-up (orange) with age, mission duration, mission experience, interval between return date and postflight MRI, interval between return date and follow-up MRI, and pre- to postflight change in grey matter volume (ΔGMV). Values for age, mission duration and mission experience are excluded to avoid subject identification.

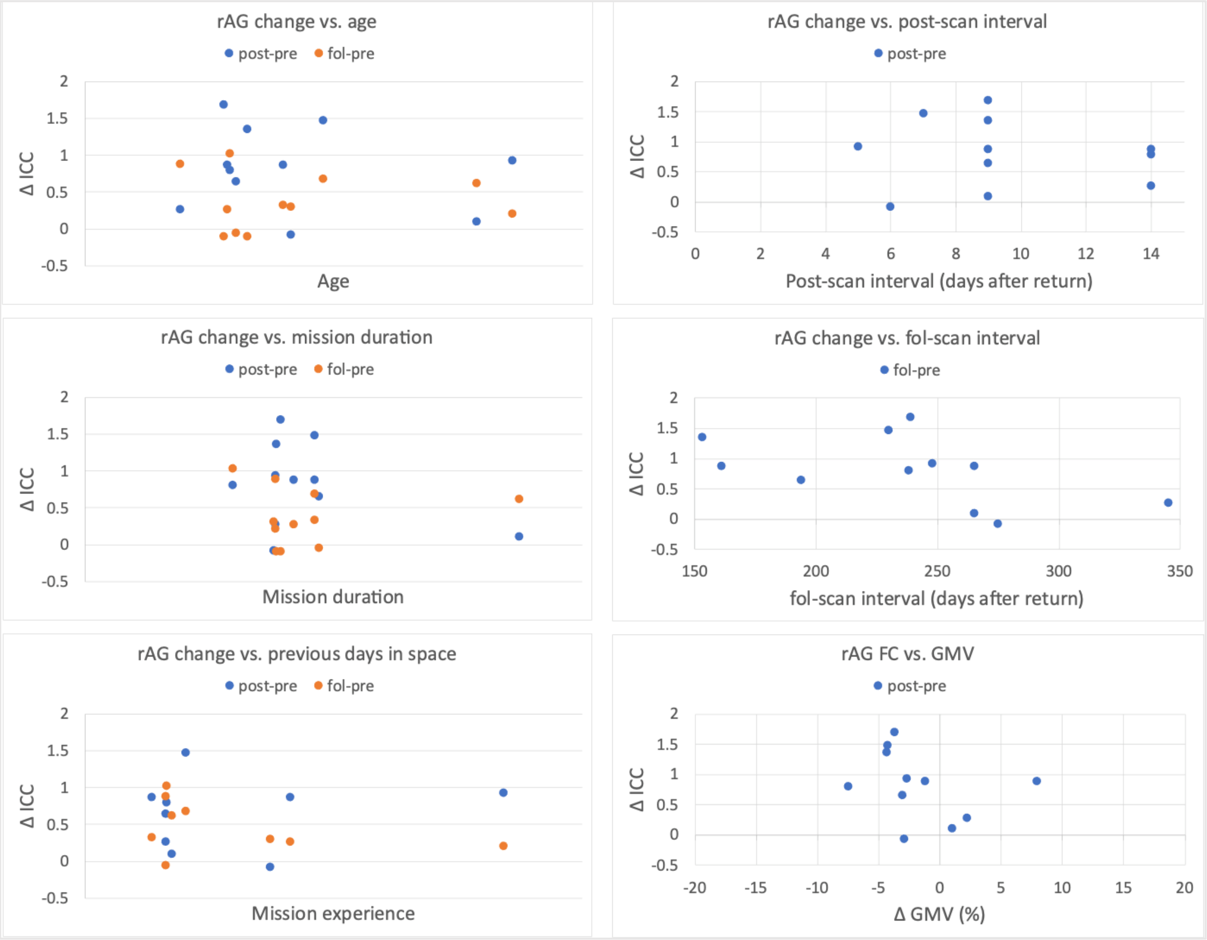

Figure S3. Connectivity change of the right angular gyrus (rAG) in function of demographic variables and grey matter volume changes. Scatterplots of the connectivity (ICC) changes from pre- to post-flight (blue) and preflight to follow-up (orange) with age, mission duration, mission experience, interval between return date and postflight MRI, interval between return date and follow-up MRI, and pre- to postflight change in grey matter volume (ΔGMV). Values for age, mission duration and mission experience are excluded to avoid subject identification.

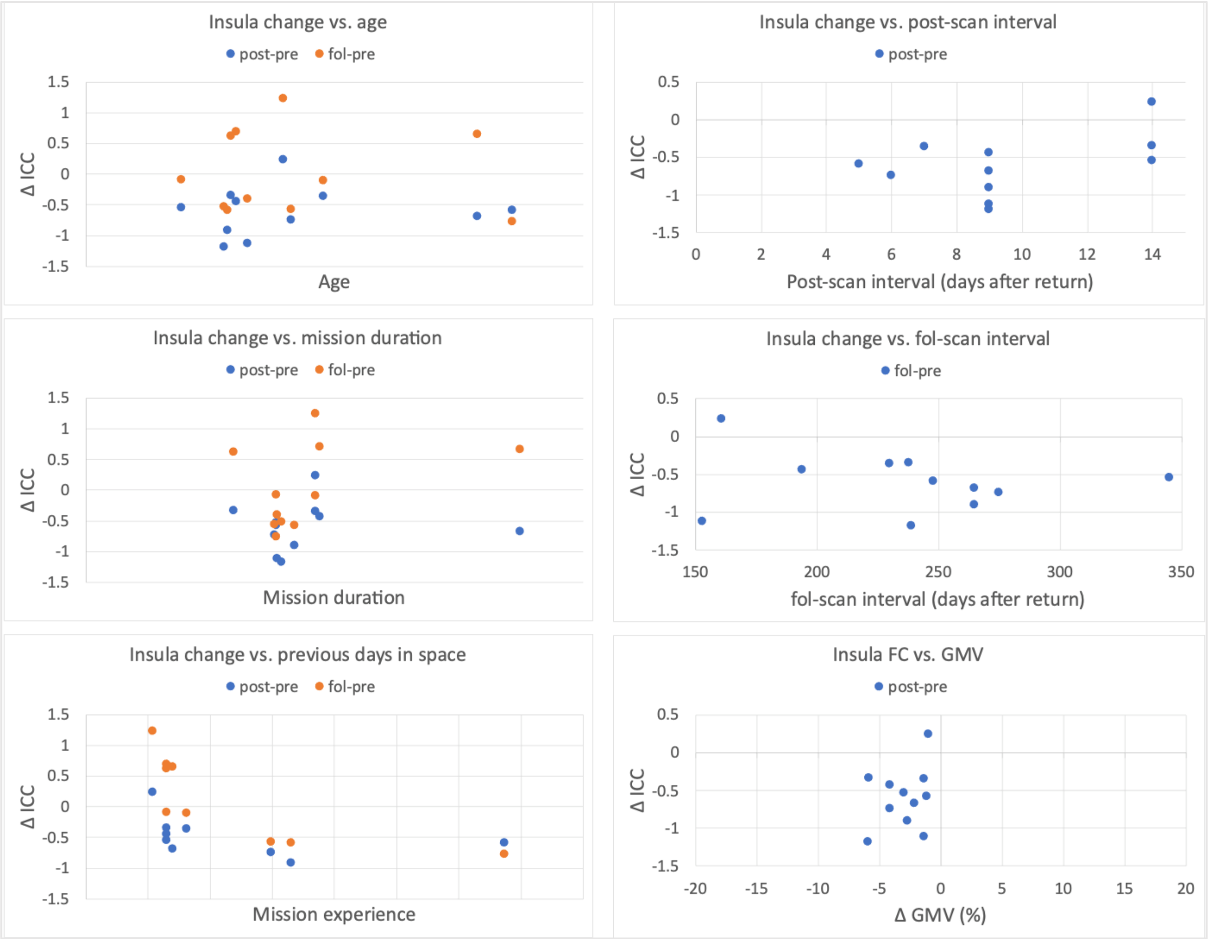

Figure S4. Connectivity change of the bilateral insula in function of demographic variables and grey matter volume changes. Scatterplots of the connectivity (ICC) changes from pre- to post-flight (blue) and preflight to follow-up (orange) with age, mission duration, mission experience, interval between return date and postflight MRI, interval between return date and follow-up MRI, and pre- to postflight change in grey matter volume (ΔGMV). Values for age, mission duration and mission experience are excluded to avoid subject identification.
