# Supplementary material for: Prolonged microgravity induces reversible and persistent changes on human cerebral connectivity
Source: Commun Biol. 2023 Jan 13;6:46. doi: 10.1038/s42003-022-04382-w (PMC9839680; doi:10.1038/s42003-022-04382-w)
Supplement: Supplementary file 2 — Description of Additional Supplementary Files [file 42003_2022_4382_MOESM2_ESM.pdf]

Description of Additional Supplementary Files

File name: Supplementary Data 1

Description: the source data behind the line graphs in figures 1, 2, and 3 and behind the scatterplots in the supplementary figures. Note that age, mission duration, and previous days in space cannot be disclosed due to anonymity reasons.
