# Supplementary material for: Prolonged microgravity induces reversible and persistent changes on human cerebral connectivity
Source: Commun Biol. 2023 Jan 13;6:46. doi: 10.1038/s42003-022-04382-w (PMC9839680; doi:10.1038/s42003-022-04382-w)
Supplement: Supplementary file 4 — Reporting summary [file 42003_2022_4382_MOESM4_ESM.pdf]

Corresponding author(s): Steven Jillings
Last updated by author(s): Nov 17, 2022

Reporting Summary

Nature Portfolio wishes to improve the reproducibility of the work that we publish. This form provides structure for consistency and transparency in reporting. For further information on Nature Portfolio policies, see our Editorial Policies and the Editorial Policy Checklist.

Statistics

For all statistical analyses, confirm that the following items are present in the figure legend, table legend, main text, or Methods section.

n/a	Confirmed	
☐	☒	The exact sample size (*n*) for each experimental group/condition, given as a discrete number and unit of measurement
☐	☒	A statement on whether measurements were taken from distinct samples or whether the same sample was measured repeatedly
☐	☒	The statistical test(s) used AND whether they are one- or two-sided<br>*Only common tests should be described solely by name; describe more complex techniques in the Methods section.*
☐	☒	A description of all covariates tested
☐	☒	A description of any assumptions or corrections, such as tests of normality and adjustment for multiple comparisons
☐	☒	A full description of the statistical parameters including central tendency (e.g. means) or other basic estimates (e.g. regression coefficient) AND variation (e.g. standard deviation) or associated estimates of uncertainty (e.g. confidence intervals)
☐	☒	For null hypothesis testing, the test statistic (e.g. *F*, *t*, *r*) with confidence intervals, effect sizes, degrees of freedom and *P* value noted<br>*Give P values as exact values whenever suitable.*
☐	☒	For Bayesian analysis, information on the choice of priors and Markov chain Monte Carlo settings
☒	☐	For hierarchical and complex designs, identification of the appropriate level for tests and full reporting of outcomes
☐	☒	Estimates of effect sizes (e.g. Cohen's *d*, Pearson's *r*), indicating how they were calculated

Our web collection on statistics for biologists contains articles on many of the points above.

Software and code

Policy information about availability of computer code

Data collection	No custom codes were used for the data collection
Data analysis	No custom codes were used for the data analysis that was central to reaching the conclusions.

For manuscripts utilizing custom algorithms or software that are central to the research but not yet described in published literature, software must be made available to editors and reviewers. We strongly encourage code deposition in a community repository (e.g. GitHub). See the Nature Portfolio guidelines for submitting code & software for further information.

Data

Policy information about availability of data

All manuscripts must include a data availability statement. This statement should provide the following information, where applicable:

- Accession codes, unique identifiers, or web links for publicly available datasets
- A description of any restrictions on data availability
- For clinical datasets or third party data, please ensure that the statement adheres to our policy

The whole-brain T-score maps for all statistical tests that were run have been uploaded to Neurovault (https://neurovault.org/collections/12152/). All extracted connectivity values from each cluster from the ICC analysis are provided as supplementary data in xlsx format. Per request, other data files may be provided as well by contacting steven.jillings@uantwerpen.be.

Human research participants

Policy information about studies involving human research participants and Sex and Gender in Research.

Reporting on sex and gender	Only male human subjects took part in our study, which is the result of the available test subjects during the course of our study. Likewise, control subjects were matched for sex and were thus also all male.
Population characteristics	The median age of the cosmonaut group was 45 years. All cosmonauts were male, which was purely determined by the ISS flight schedule. All cosmonauts engaged in ISS missions with a median mission duration of 173 days.
Recruitment	Cosmonauts were recruited through the European Space Agency (ESA) and Roscosmos. The cosmonauts participate on a voluntary basis. All cosmonauts have the chance to participate to our study.
Ethics oversight	The study was approved by the Institutional Review Board of the Antwerp University Hospital (13/38/357), the European Space Agency (ESA) Medical Board, the Committee of Biomedicine Ethics of the Institute of Biomedical Problems of the Russian Academy of Science, and the Human Research Multilateral Review Board. All participants provided a signed informed consent and all investigations were performed in accordance with the principles listed in the Declaration of Helsinki and its amendments.

Note that full information on the approval of the study protocol must also be provided in the manuscript.

Field-specific reporting

Please select the one below that is the best fit for your research. If you are not sure, read the appropriate sections before making your selection.

☒ Life sciences ☐ Behavioural & social sciences ☐ Ecological, evolutionary & environmental sciences

For a reference copy of the document with all sections, see nature.com/documents/nr-reporting-summary-flat.pdf

Life sciences study design

All studies must disclose on these points even when the disclosure is negative.

Sample size	15 cosmonauts were included in the study, who were tested twice (preflight and postflight). 11 of these cosmonauts were also tested a third time (follow-up). The data drop-out was due to voluntarily quitting the study for 3 cosmonauts, and a pending followup measurement for 1 cosmonaut. 14 control subjects were also included in the study, who were tested twice with a similar time interval as the duration of the space mission. Sample size was determined by having at least 10 participants scanned at each of the three time points.
Data exclusions	Only participants who were tested at least at preflight and postflight were included. No data were excluded after this initial selection criterion.
Replication	Due to the logistic difficulties and the unique setting in which data from cosmonauts are acquired, we were not able to perform replication analyses.
Randomization	Groups were predetermined by having cosmonauts who engaged in missions to the International Space Station in one group, and control participants who stayed on Earth in another group.
Blinding	No blinding procedure was implemented in this study. The results of this study were not dependent on subjective assessments.

Reporting for specific materials, systems and methods

We require information from authors about some types of materials, experimental systems and methods used in many studies. Here, indicate whether each material, system or method listed is relevant to your study. If you are not sure if a list item applies to your research, read the appropriate section before selecting a response.

Materials & experimental systems

n/a	Involved in the study
☒	☐ Antibodies
☒	☐ Eukaryotic cell lines
☒	☐ Palaeontology and archaeology
☒	☐ Animals and other organisms
☒	☐ Clinical data
☒	☐ Dual use research of concern

Methods

n/a	Involved in the study
☒	☐ ChIP-seq
☒	☐ Flow cytometry
☐	☒ MRI-based neuroimaging

Magnetic resonance imaging

Experimental design

Design type	resting-state
Design specifications	300 continuous resting-state measurements
Behavioral performance measures	N/A

Acquisition

Imaging type(s)	functional
Field strength	3T
Sequence & imaging parameters	gradient echo; EPI, FOV=192x192x126; matrix size: 64x64x42, slice thickness: 3mm; orientation: axial; TE=30ms; TR=2000ms; flip angle: 77°
Area of acquisition	whole-brain acquisition

Diffusion MRI ☐ Used ☒ Not used

Preprocessing

Preprocessing software	SPM12 (revision 7219), CONN (v18)<br>preprocessing steps:<br>1) Slice timing correction<br>2) motion correction<br>3) segmentation of T1w images<br>4) smoothing of EPI images by a Gaussian 3D kernel of 6mm FWHM<br>5) outlier detection using the artifact removal toolbox (ART)
Normalization	Data were non-linearly normalized to MNI space. Estimation of native to MNI space transformation was performed using anatomical images of 1mm isotropic voxels. Functional images were warped into MNI space after affine coregistration to the anatomical image in native space and using the deformation fields resulting from the segmentation step.
Normalization template	Data were normalized to MNI space
Noise and artifact removal	Noise covariates included:<br>1) Realignment parameters resulting from the motion correction step + their first temporal derivatives<br>2) Outlier volumes resulting from ART (scrubbing)<br>3) Signal from white matter (WM) and cerebrospinal fluid (CSF) (based on anatomical scan segmentation) using aCompCor. The 10 first principal components of both WM and CSF were entered as nuisance regressors.<br>4) Linear detrending<br>5) Bandpass filtering of 0.008 to 0.09 Hz
Volume censoring	N/A

Statistical modeling & inference

Model type and settings	Mass univariate analysis, followed by cluster-wise inference.<br>First-level model: A voxel-by-voxel correlation matrix is computed, followed by a calculation of the intrinsic connectivity contrast (ICC) in each voxel i of the brain. The ICC reflects the degree to which voxel i is correlated to each other voxel in the brain. All correlation values were first normalized to have a mean of 0 and unit standard deviation.<br>Second-level: fixed effects
Effect(s) tested	No tasks involved in this study

Specify type of analysis: ☐ Whole brain ☐ ROI-based ☒ Both

Anatomical location(s)	Resulting clusters from the ICC analysis were subsequently used as ROIs to map out the regions with which the ROI exhibits altered connectivity (ICC is a measure of global connectivity. Hence, a post-hoc seed-to-voxel test was performed to elaborate on specific region-to-region connectivity changes that encompass the global connectivity change).
Statistic type for inference<br>(See Eklund et al. 2016)	For evaluating connectivity changes in time: voxel-level whole-brain threshold of p<0.005, followed by a cluster-level threshold of p<0.05; parametric tests<br>For evaluating time-independent network association of the ROIs: voxel-level whole-brain threshold of p<0.001, followed by a cluster-level threshold of p<0.05; parametric tests

Correction	At the cluster-level, correction for multiple comparisons was applied using FWE (p<0.05). No correction was performed for the initial voxel-level threshold (p<0.005).

Models & analysis

n/a	Involved in the study
☐	☒ Functional and/or effective connectivity
☒	☐ Graph analysis
☒	☐ Multivariate modeling or predictive analysis

Functional and/or effective connectivity	Pearson's correlation coefficients
